# Bacteroides thetaiotaomicron, a Model Gastrointestinal Tract Species, Prefers Heme as an Iron Source, Yields Protoporphyrin IX as a Product, and Acts as a Heme Reservoir

**DOI:** 10.1128/spectrum.04815-22

**Published:** 2023-03-02

**Authors:** Margaux M. Meslé, Chase R. Gray, Mensur Dlakić, Jennifer L. DuBois

**Affiliations:** a Department of Chemistry and Biochemistry, Montana State University, Bozeman, Montana, USA; b Department of Microbiology and Cell Biology, Montana State University, Bozeman, Montana, USA; University of Minnesota Twin Cities

**Keywords:** dietary iron, human gut, microbial metabolism, commensal bacteria, porphyrins, heme, anaerobe, protoporphyrin IX, human microbiome, iron metabolism, porphyrin

## Abstract

Members of the phylum *Bacteroidetes* are abundant in healthy gastrointestinal (GI) tract flora. Bacteroides thetaiotaomicron is a commensal heme auxotroph and representative of this group. *Bacteroidetes* are sensitive to host dietary iron restriction but proliferate in heme-rich environments that are also associated with colon cancer. We hypothesized that B. thetaiotaomicron may act as a host reservoir for iron and/or heme. In this study, we defined growth-promoting quantities of iron for B. thetaiotaomicron. B. thetaiotaomicron preferentially consumed and hyperaccumulated iron in the form of heme when presented both heme and nonheme iron sources in excess of its growth needs, leading to an estimated 3.6 to 8.4 mg iron in a model GI tract microbiome consisting solely of B. thetaiotaomicron. Protoporphyrin IX was identified as an organic coproduct of heme metabolism, consistent with anaerobic removal of iron from the heme leaving the intact tetrapyrrole as the observed product. Notably, no predicted or discernible pathway for protoporphyrin IX generation exists in B. thetaiotaomicron. Heme metabolism in congeners of B. thetaiotaomicron has previously been associated with the 6-gene *hmu* operon, based on genetic studies. A bioinformatics survey demonstrated that the intact operon is widespread in but confined to members of the *Bacteroidetes* phylum and ubiquitous in healthy human GI tract flora. Anaerobic heme metabolism by commensal *Bacteroidetes* via *hmu* is likely a major contributor to human host metabolism of the heme from dietary red meat and a driver for the selective growth of these species in the GI tract consortium.

**IMPORTANCE** Research on bacterial iron metabolism has historically focused on the host-pathogen relationship, where the host suppresses pathogen growth by cutting off access to iron. Less is known about how host iron is shared with bacterial species that live commensally in the anaerobic human GI tract, typified by members of phylum *Bacteroidetes*. While many facultative pathogens avidly produce and consume heme iron, most GI tract anaerobes are heme auxotrophs whose metabolic preferences we aimed to describe. Understanding iron metabolism by model microbiome species like Bacteroides thetaiotaomicron is essential for modeling the ecology of the GI tract, which serves the long-term biomedical goals of manipulating the microbiome to facilitate host metabolism of iron and remediate dysbiosis and associated pathologies (e.g., inflammation and cancer).

## INTRODUCTION

Iron is an essential nutrient and growth-limiting element for many organisms and cells and at the same time a proinflammatory agent (1–3). Within the human body, most iron forms ferrous or ferric complexes with protoporphyrin IX (PPIX) (1–3), referred to here simply as “heme,” which plays numerous roles in the transport and storage of both O_2_ and electrons and in catalysis. Sequestration of growth-promoting iron in its heme and nonheme forms occurs as part of the innate immune response in humans and animals, following the detection of invading pathogens (2, 4). Both environmental and pathogenic microbes, including well-studied members of the Pseudomonas, *Porphyromonas*, and Staphylococcus genera, have consequently evolved a variety of mechanisms for liberating and assimilating diverse chemical species of heme and nonheme iron (2, 3, 5).

The community of microbes in the healthy human gastrointestinal (GI) tract, in contrast, lives under conditions where the host must permit sharing of iron resources and where the anaerobic, neutral to acidic environment is expected to stabilize iron in either its water-soluble Fe(II) or heme form. The latter is relatively chemically stable and expected to survive intact in much of the GI tract and into the colon (1, 2, 4). Additional heme is also available from cells in the continuously regenerating intestinal lining, where macrophage involvement in recycling the heme is not expected. Aqueous Fe(II) [Fe(II)_aq_] is transported into many bacterial cells by FeoAB, a member of the ABC (ATP-binding cassette) family of metal transporters, making Fe(II)_aq_ a hypothetically attractive principal source of iron for GI tract communities. However, some of the most abundant flora in the gut are heme auxotrophic anaerobic bacteria, including members of the *Bacteroidetes* phylum, which require intact heme for their survival but do not possess known pathways for biosynthesis of heme or PPIX (1, 2, 4, 5).

Where this heme comes from is not completely clear. While the hemoglobin from lysed red blood cells represents a rich source of heme for hemolytic pathogens, heme and nonheme iron must be supplied to commensal species in ways that would not excite an immune response: by the host diet directly, scavenged from host intestinal cells during cell turnover, or synthesized by other heme-heterotrophic species in the consortium (2, 3, 5). These heme heterotrophs are typified by enterobacteria like Escherichia coli, which are metabolically capable of heme biosynthesis but generally low in abundance. Given their dependence on their environment for essential heme, we hypothesized that *Bacteroides* species would be particularly sensitive to disruptions in their community structures or iron supplies. In prior work, we showed that iron-challenged mice underwent dramatic changes in the composition of their microbiota (6). Heme auxotrophic members of the *Bacteroidales* order and *Porphyromonadaceae* family were highly sensitive to host iron starvation, becoming irreversibly lost from the mouse microbiome (6).

In contrast with facultative pathogens, less is understood about how anaerobic commensal species, and especially heme auxotrophic bacteria, meet their iron requirements, and how their iron metabolic activities are influenced by other members of the GI tract consortia and the host (2, 3, 5). Notably, the most biologically common mechanisms for metabolizing heme to liberate nonheme iron use O_2_-dependent heme oxygenases (7). Two anaerobic mechanisms for releasing heme iron from PPIX have been described, encoded by the Escherichia coli heme uptake (*chu*) operon, long associated with hemorrhagic/uropathogenic strains (8), and the heme metabolism uptake (*hmu*) operon (1, 3). Bacteroides thetaiotaomicron strain VPI 5482 (ATCC 29148) is a nonpathogenic Gram-negative heme auxotroph (9–11) that has been used as a model for GI tract commensals (1, 10). It possesses an apparently intact *hmu* operon, encoding a heme transporter and the CobN homolog HmuS, where CobN is part of a protein complex responsible for inserting Co(II) into a cobalamin precursor in the aerobic cobalamin (vitamin B_12_) biosynthetic pathway. Related ChlH/BchH proteins are part of chlorophyll/bacteriochlorophyll biosynthetic pathways, where they catalyze Mg^2+^ into the protoporphyrin IX macrocycle (12). Notably, B. thetaiotaomicron is also unable to biosynthesize cobalamin, though proteins involved in its uptake and use have been described (13). Prior work with a pathogenic strain of Bacteroides fragilis unequivocally demonstrated that the HmuS protein is involved in the removal of iron from heme, though its biochemical role is not understood (4). B. thetaiotaomicron also possesses genes encoding SufB and SufC, necessary and sufficient for building FeS clusters (14), which are essential for many electron transfer processes and especially abundant in anaerobic species.

Given the need for and availability of both heme and nonheme sources of iron for GI tract species, we were interested in whether one is preferentially assimilated by B. thetaiotaomicron and in the resulting distribution of heme and nonheme iron within the cell. We were moreover interested in examining whether B. thetaiotaomicron would exhibit differences in its iron metabolism behavior relative to pathogenic B. fragilis strains (4), for example, in heme and nonheme iron source preference or accumulation. In this study, we tested the hypothesis that B. thetaiotaomicron would be able to survive with heme as a sole source of iron based on the presence of an annotated *hmu* operon and previously proposed roles of its gene products in heme metabolism (1). We quantified its heme and nonheme iron requirements, its heme accumulation, and its porphyrin production, using culture-based assays and targeted metabolite analyses. Using bioinformatics, we examined the composition and distribution of *hmu* operons among different groups of bacteria, including an extensive and diverse set of *Bacteroidetes* species. We used both analyses to estimate the iron store represented by similar heme-auxotrophic bacteria in the human GI tract and to refine the hypothetical model for *hmu* operon function. Taken together, our results suggest that these commensal heme auxotrophs are avid heme accumulators. Bacteria like these likely represent a significant iron reservoir in the host GI tract, maintaining heme and Fe homeostasis and acting as a pro-oxidant source.

## RESULTS AND DISCUSSION

### A model commensal strain was selected.

A nonpathogenic strain of Bacteroides thetaiotaomicron was purchased from the American Type Culture Collection (ATCC 29148) corresponding to the strain VPI 5482, for which the genome has been fully sequenced (1). The freeze-dried stock cells were resuspended and imaged, their identities were confirmed by PCR and microscopic imaging, and glycerol stocks were generated as described in detail in the supplemental material (see supplemental text, Fig. S1 and S2, and Tables S1 and S2).

### Growth-promoting heme and nonheme iron concentrations were defined.

**FIG 1:**
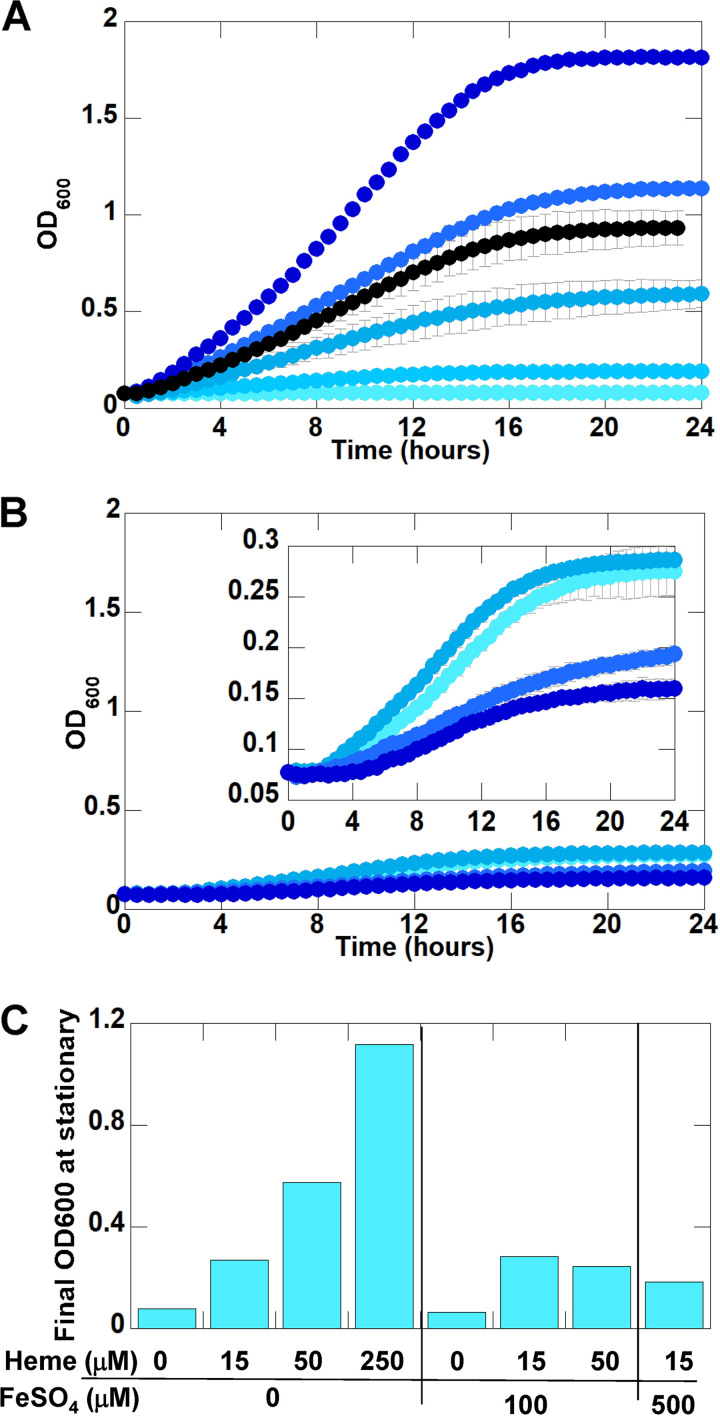
B. thetaiotaomicron grows optimally in minimal medium with ≤0.5 mM hemin and no FeSO_4_ supplementation and diminishes with increasing hemin. (A) Concentrations of heme iron were increased in the absence of nonheme iron. Heme concentrations were 0 (light blue), 15, 50, 100, 250, and 500 μM (dark blue). The black curve represents 1 mM heme iron. (B) Concentrations of nonheme iron were increased in the presence of 15 μM heme iron (typical of *Bacteroides* media). The inset highlights FeSO_4_ concentrations of 0 μM (light blue), 100, 500, and 1,000 μM (dark blue). (C) Comparison of final OD_600_ at stationary phase (24 h) for B. thetaiotaomicron strain VPI 5482 grown with various concentrations of heme and nonheme iron. One-way analysis of variance (ANOVA) was performed, with nonpaired multiple comparison and comparison to the mean of the control (0 μM heme and 0 μM iron). The analysis indicates statistical difference from the control data, as well as between compared samples.

Preferential use and growth-promoting concentrations of heme and nonheme iron were examined by comparing B. thetaiotaomicron cultures grown in a rich, beef broth medium (15, 16) and a chemically defined, iron-free minimal medium (MM) (15–17) supplemented with concentrations of heme and/or ferrous sulfate (FeSO_4_). Experiments were carried out in a glove box with an atmosphere composed of 2.5% H_2_ in N_2_ (no CO_2_, H_2_ concentration subflammable), using an automated plate reader to screen for growth efficiency under a broad range of heme (Fig. 1A) and nonheme (Fig. 1B) iron concentrations. Culture growth was monitored via optical density at 600 nm (OD_600_) over time.

In chemically defined minimal media, B. thetaiotaomicron growth was enhanced by increasing the heme concentration, in a background of no FeSO_4_, up to 500 μM added hemin (Fig. 1A, light to dark blue curves). Added heme at ≥1 mM was apparently toxic, as illustrated by both slower growth and lower concentrations of cells at saturation (black curve). In cultures supplemented with 15 μM heme (Fig. 1B inset, lighter blue curves), a concentration typical of several types of media used for members of the *Bacteroides* genus (15–17), the B. thetaiotaomicron growth rate was not enhanced by addition of up to 100 μM nonheme iron. Addition of ≥100 μM FeSO_4_ was toxic (darker blue curves). Final OD_600_ values at stationary phase (24 h) demonstrated analogous trends (Fig. 1C), where hemin concentrations up to 0.5 mM led to greater cell density while addition of nonheme iron up to 100 μM had no effect. We therefore used ≤500 μM heme, without added iron sulfate, as the approximate range of growth-promoting iron for the defined medium.

### Nonheme iron did not enhance the growth of B. thetaiotaomicron in scaled-up cultures grown under an optimal atmosphere.

B. thetaiotaomicron was subsequently grown in scaled-up, triplicate biological assays in anaerobic culture tubes (10 mL) with a headspace atmosphere composed of 10% H_2_ (a source of electrons for anaerobic growth) and 5% CO_2_ in N_2_, canonical for this species (15–17). These cultures were grown to stationary phase, collected by centrifugation, and analyzed for iron metabolites (Table 1). A defined minimal medium supplemented with either 15 μM heme iron or both 15 μM hemin and 15 μM FeSO_4_ was used; 15 μM was chosen because it is both within the growth-promoting range identified in Fig. 1 and typical of defined media used with this genus (15–17). A medium containing 1 μM hemin added to beef broth (15), a rich medium used to revive *Bacteroides* species (15, 16), provided a growth control for the experiments. The as-prepared beef broth contained ≤1 μM heme iron and ~20 μM nonheme iron. The as-prepared minimal medium contained ≤1 μM total iron prior to amendment with hemin and/or FeSO_4_ (data not shown).

**TABLE 1:** B. thetaiotaomicron cells acquire heme and nonheme iron and preferentially accumulate heme^*a*^

Growth condition^*b*^	Total iron (pmol/μg DNA)	Heme (pmol/μg DNA)	Heme (total iron) (%)	PPIX (pmol/μg DNA)
Rich medium	254 ± 76	1.59 ± 0.47	1	0.44 ± 0.48
MM + 15 μM hemin	380 ± 64	140 ± 13	37	1.35 ± 0.45
MM + 15 μM FeSO_4_ + 15 μM hemin	584 ± 55	120 ± 39	20	1.23 ± 0.91

a Intracellular concentrations of total iron, heme, and PPIX concentrations at stationary phase in B. thetaiotaomicron cells are shown. Values were measured in nanomoles per milliliter of culture via AA and LC-MS and further normalized to the corresponding amount of DNA extracted from the pellet of 1 mL culture (picomoles per microgram of DNA), where 1 μg DNA is the amount of genetic material in 1.48 × 10^8^ B. thetaiotaomicron cells. Values are the means ± SD of 3 biological replicates and 3 technical replicates (*n* = 9).

b MM, minimal medium (≤1 μM total iron). Rich medium (beef broth) contained ≤1 μM heme iron and ~20 μM nonheme iron.

**FIG 2:**
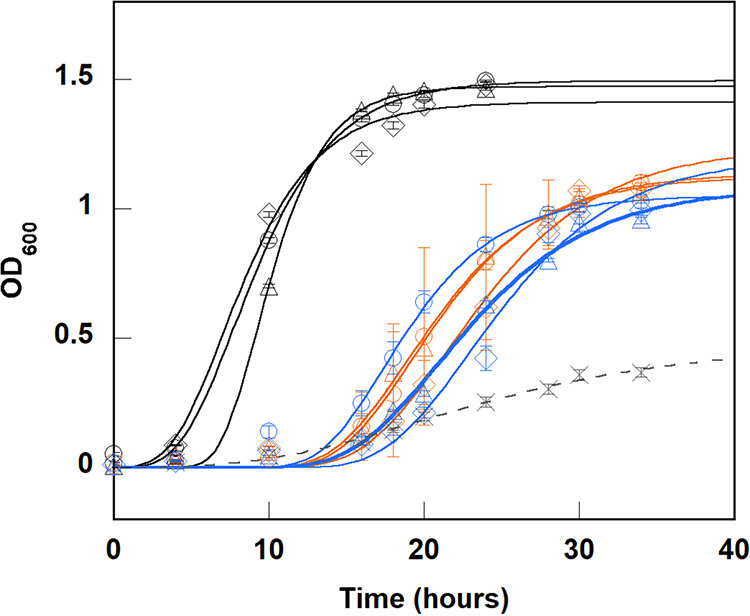
B. thetaiotaomicron growth was not enhanced by added nonheme iron. Growth of B. thetaiotaomicron strain VPI 5482 in rich medium (beef broth [black]) and defined minimal medium supplemented with 15 μM heme in the presence (orange) or absence (blue, dashed lines) of 15 μM nonheme iron (FeSO_4_) is shown. The symbols (circles, diamonds, and triangles) represent 3 biological replicate assays. A control without iron (X, gray) was also performed. The error bars indicate ±1 standard deviation for triplicate biological assays and were smaller than the symbols used for the rich medium plots. Curves were fit to the Gompertz equation for exponential rise (see Materials and Methods). Growth was assessed by optical density measurements at 600 nm, and the corresponding DNA concentration was calculated using the correlation factor determined from data in Table S3 (6 μg mL^−1 ^au^−1^).

The fastest growth was observed in the rich medium as expected, reaching stationary phase and saturating at an OD_600_ of 1.46 ± 0.043 after a latency period of 4.94 ± 0.17 h (Fig. 2). The maximal growth rate (μ_m_) during the exponential phase was determined as 0.260 ± 0.061 au h^−1^ (where au is absorbance unit at 600 nm), or 2.34 (±0.55) × 10^8^ cells h^−1^ (Table S1), by fitting the growth curves to a modified Gompertz equation describing bacterial growth (18). More robust growth in rich versus the defined media may be explained by the availability of micronutrients and metabolism of complex carbohydrates from the beef broth, as B. thetaiotaomicron is known to hydrolyze dietary polysaccharides that are otherwise unmetabolizable by animal hosts (11, 19–23). Growth in the defined medium supplemented with 15 μM heme only, or 15 μM heme plus 15 μM nonheme iron, yielded lag times, saturation yields, and maximal growth rates that were each the same within error (Fig. 2 and Table S2). A latency period of about 15 h was observed under both defined and minimal medium conditions, after which the cells grew exponentially until 30 to 34 h, when they reached stationary phase. Comparatively, cells grown under defined iron conditions had less than half the maximal growth rate (~0.1 au h^−1^, or ~1 × 10^8^ cells h^−1^) of those grown in rich media and slightly lower cell numbers (~75% of rich media) at saturation (Table S1). Little growth was observed under the control condition with no added iron. Together, these experiments confirmed that added nonheme iron has little effect on B. thetaiotaomicron growth under canonical conditions of headspace and heme concentration. This result is in contrast to prior work with a pathogenic, clinically isolated strain of B. fragilis, which appeared to obtain a growth benefit from cosupply of heme and nonheme iron (FeSO_4_) (4). Because the growth conditions used in this and the previously published study were similar, we speculate that the greater bioavailability of Fe(II) to the pathogenic strain is genetically controlled and supportive of a pathogenic lifestyle. These experiments also produced biomass for analyses aimed at quantifying their iron metabolites and relating these to cell numbers (see below).

The cells exhibited some tendency to clump and grew at inconsistent rates on solid plate media, particularly when cultivated in the presence of added hemin. We consequently examined DNA concentration and cell pellet mass as alternative means for monitoring cell growth (see the supplemental material). Optical density correlated well with both pelleted cell masses (3.4 mg cell mass mL culture^−1 ^au^−1^) and DNA concentrations (6.0 μg DNA mL culture^−1 ^au^−1^) with high reproducibility (Fig. S2 and Table S3). Based on the 6.26-Mbp size of the B. thetaiotaomicron VPI 5482 genome (7, 23), the approximate mass of DNA per cell is 6.76 fg; or, alternatively, 1.48 × 10^8^ B. thetaiotaomicron cells possess a total of 1 μg genomic DNA. Hence, under conditions used in this study, an OD_600_ of 1 corresponded to 6.0 μg DNA, 9.0 × 10^8^ B. thetaiotaomicron cells (equivalent to CFU), or 3.4 mg wet cell paste mL culture^−1^. These conversion factors were used to relate the measured optical densities and metabolite concentrations (see below) to cellular quantities.

### Total concentrations of Fe acquired by B. thetaiotaomicron were not strongly dependent on the growth medium.

To quantify iron metabolic activity by B. thetaiotaomicron, the freshly saturated cultures monitored in Fig. 2 were first pelleted and lysed. The total iron content of the soluble, intracellular fraction was quantified via atomic absorption spectroscopy (AA) (Table 1) and normalized per microgram of genomic DNA (see supplemental text, Tables S2 and S3, and Fig. S2). Intracellular iron increased only slightly in proportion to the total amount of iron in the medium, measuring 2 to 3 μmol iron L^−1^ saturated cell culture, or 250 to 580 pmol per μg genomic DNA, where 1 μg represents the amount of genomic DNA in roughly 1.3 × 10^8^ cells. Similar cellular iron concentrations were observed despite differences in the chemical speciation (all heme versus heme plus nonheme sources) and concentration of iron (1 to 30 μM total) in the 3 media. This suggests that iron and/or heme uptake is regulated by the cell so that a homeostatic concentration is maintained. To further explore this observation, the chemical speciation (heme versus nonheme) of the intracellular iron pools was subsequently quantified for cells grown under each condition from Fig. 2.

### B. thetaiotaomicron grown under nutrient limitation accumulates heme.

**FIG 3:**
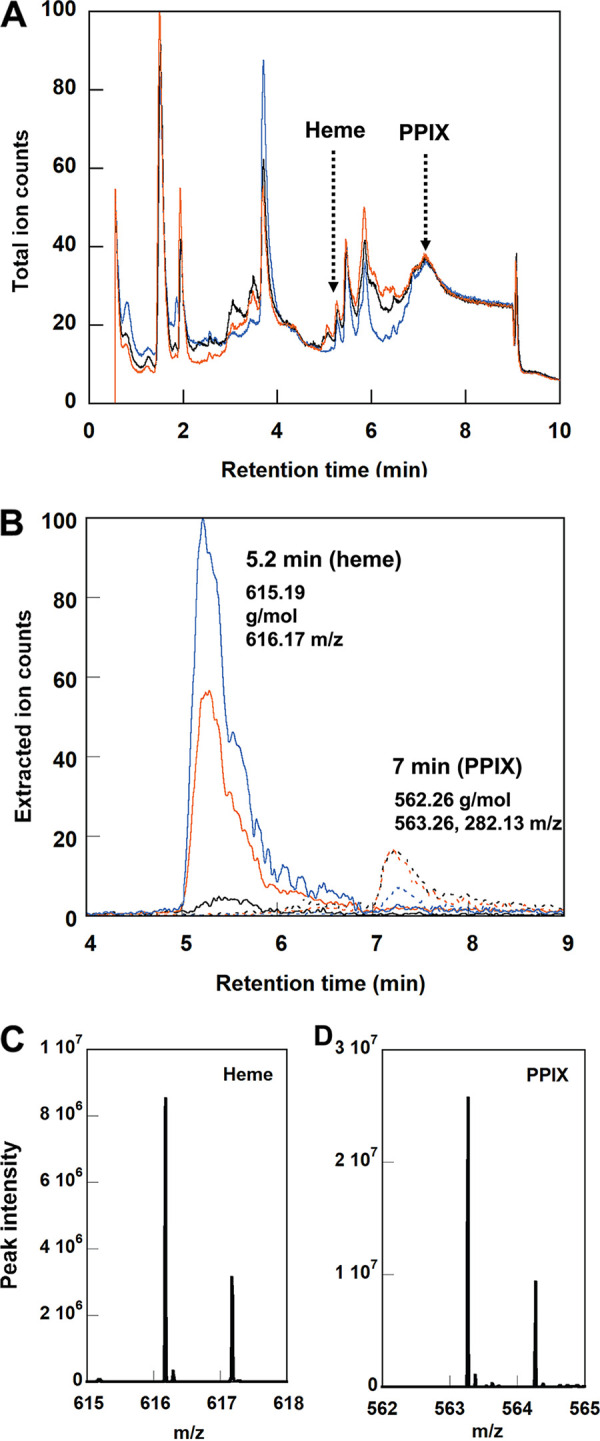
Quantification of heme and PPIX metabolites in B. thetaiotaomicron cells. (A) Representative total ion chromatogram (TIC) showing metabolites extracted from B. thetaiotaomicron grown in rich media. (B) Extracted ion chromatograms (EICs) illustrate heme and PPIX detected via the predicted exact masses of heme (solid lines) and PPIX (dashed lines) and quantified by integrating peak areas (relative to standards). (C) Representative mass spectrum of heme. (D) Representative mass spectrum of PPIX. Metabolites were isolated from 10-mL cell cultures grown in rich medium (black), minimal medium plus 15 μM hemin (blue), and minimal medium plus 15 mM hemin and 15 mM FeSO_4_ (orange) (37°C; 85% N_2_, 10% H_2_, and 5% CO_2_ in the headspace). The molecular weight, *m/z* value, and elution times for each compound are reported in panel B.

The heme and porphyrin species from Fig. 2 were concentrated on solid-phase chromatography media prior to analysis (see supplemental text and Fig. S3). Heme and PPIX were concurrently quantified using liquid chromatography coupled to mass spectrometry (LC-MS) (Table 1 and Fig. 3). The cell samples revealed distinct peaks for heme (616.17 mass per charge [*m/z*]) and PPIX (563.26 *m/z*, with a 282.13 *m/z* fragment) which were discernible in the complex, total ion chromatograms (TICs) (Fig. 3A). The extracted ion chromatograms (EICs) (Fig. 3B) for these ions were integrated and used to quantify each metabolite relative to concentration standards (Fig. S4). Very little intracellular heme was detected for cells grown in the rich medium (~1.6 pmol/μg DNA), while comparable and ~100-fold-higher heme concentrations were observed under the two defined medium conditions (120 to 140 pmol/μg DNA), where heme was available at 15 μM in each case (Table 1). In comparison, the rich medium cultures were initially supplemented with only 1 μM heme. These LCMS results suggest that heme accumulated within the cells grown in the minimal media (15–17).

Notably, even when 15 μM concentrations of both heme and nonheme iron were supplied, heme was nonetheless preferentially incorporated, such that 20% of the cellular iron in either case was present as heme. Similar preferential use of heme iron has been observed for pathogenic strains of Staphylococcus aureus, demonstrating its adaptation to red blood cells as an abundant iron source (24). The fact that B. thetaiotaomicron cells supplemented solely with heme grew and contained nonheme iron confirmed that B. thetaiotaomicron metabolized heme to release its iron. The expected initial organic by-product of heme metabolism (PPIX) was also quantified in B. thetaiotaomicron cells (Fig. 3 and Table 1), though in much smaller amounts. This suggested that some of the heme incorporated by B. thetaiotaomicron was metabolized to yield PPIX and free iron, consistent with the presence of an *hmu* operon putatively encoding proteins for the uptake, trafficking, and removal of the iron from heme (1, 25, 26). On average, cells grown in minimal media containing 15 μM hemin, with or without added FeSO_4_, exhibited comparable intracellular PPIX concentrations within error (1 to 2 pmol/μg DNA). Cells grown in rich medium had a slightly lower PPIX concentration on average (0.4 pmol/μg DNA). This result suggests that cells under all 3 conditions actively metabolized some amount of the incorporated heme, allowing PPIX to accrue to detectable levels before it was expelled or further metabolized.

### The microbiome serves as a significant reservoir of iron for the host.

Prior work with mice on a defined iron diet showed that with an intact microbiome, the animals withstood a 2-week iron challenge without changes to their serum iron levels and without behavioral indicators of iron stress (6). We attributed this to iron recycling from damaged red blood cells and intestinal epithelial cells. We also hypothesized that the mice may have been buffered from iron starvation by using their GI tract flora as a metabolizable source of iron. Iron from members of the *Bacteroides* family and genus that were extinguished from the microbiome as a result of the challenge may have been reabsorbed by the host. We examined this hypothesis in light of the iron levels measured in the B. thetaiotaomicron cells in the present study, given both the strong influence of iron starvation on members of the *Bacteroides* genus and the observation that *Bacteroidetes* and/or *Firmicutes* constitute 80% of a typical healthy microbiome (27, 28). We therefore estimated the iron content of a hypothetical microbiome consisting wholly of B. thetaiotaomicron as a surrogate for a *Bacteroidetes*-rich consortium. We note first that most of the human microbiome is located in the GI tract and that most of these organisms are located in the colon (29, 30). Total cells in the GI tract microbiome of a reference 70-kg man have been estimated at 3.8 × 10^13^ (30), assuming an average gut bacterium mass to be 5 pg (wet weight) (30). These numbers give a total mass of approximately 200 g for the reference human gut microbiome (0.3% of the total body weight) (30). Using the iron values measured here in picmoles per microgram of DNA (Table 1), the known size of the B. thetaiotaomicron VPI 5482 genome (7, 23), and the assumption that the total microbiome mass consisted of B. thetaiotaomicron, the total mass of microbiome-based iron was estimated at 3.6 to 8.4 mg. This range encompasses cells grown on both the rich and chemically defined media and may contain heme iron in proportion to the amount of heme available to the GI tract consortium (Table 2). A typical daily diet for an adult human contains 10 to 20 mg iron, from which 1 to 3 mg day^−1^ is absorbed, replacing the average iron loss of 0.9 to 1.0 mg day^−1^ out of the ~3 g total iron in the body (31, 32). A microbial reservoir of 3.6 to 8.4 mg iron might therefore offer several days’ worth of a buffer against iron starvation.

**TABLE 2:** A microbiome consisting solely of B. thetaiotaomicron cells (3.80 × 10^13^) offers a substantial reservoir of iron to the host^*a*^

Growth condition^*b*^	Total iron per microbiome (mg)	Total heme per microbiome (mg)
Rich medium	3.6 ± 0.2	0.020 ± 0.0
MM + 15 μM hemin	5.5 ± 0.3	2.0 ± 0.2
MM + 15 μM FeSO_4_ + 15 μM hemin	8.4 ± 0.3	1.7 ± 0.03

a Extrapolated values of total Fe in the microbiome, based on measured values for B. thetaiotaomicron normalized to DNA amounts. The calculations assume B. thetaiotaomicron as the only member of the gut microbiome and are based on published estimates of 3.80 × 10^13^ microbial cells in the colon or 200 g as the total mass (30). Mass of iron per total genomic DNA was estimated using the data in Table 1.

b Minimal medium contained ≤1 μM total iron. Rich medium (beef broth) contained ≤1 μM heme iron and ~20 μM nonheme iron.

### Informatics analyses of the *hmu* operon support possible roles predicted by prior experimental work for several of its genes.

Heme uptake and degradation have been well studied for important (facultative) aerobic human pathogens, including Pseudomonas aeruginosa, Staphylococcus aureus, and Mycobacterium tuberculosis (2, 3, 33, 34). These organisms use structurally diverse hemophores to capture and recruit heme from, for example, hemoglobin, an abundant blood-borne source of heme (35). The heme is then trafficked through the cell membrane/outer wall and into the cytoplasm, where O_2_ is used to oxidatively cleave the PPIX ligand, releasing the iron (34, 35). Some components of this system would be expected to be retained in B. thetaiotaomicron, particularly the TonB-dependent receptor/transporter system that uses the transmembrane potential gradient to drive ligand import from the periplasm and into the cytoplasm of Gram-negative bacteria. However, as an anaerobe, B. thetaiotaomicron clearly cannot make use of an O_2_-dependent mechanism for release of essential iron from PPIX.

**FIG 4:**
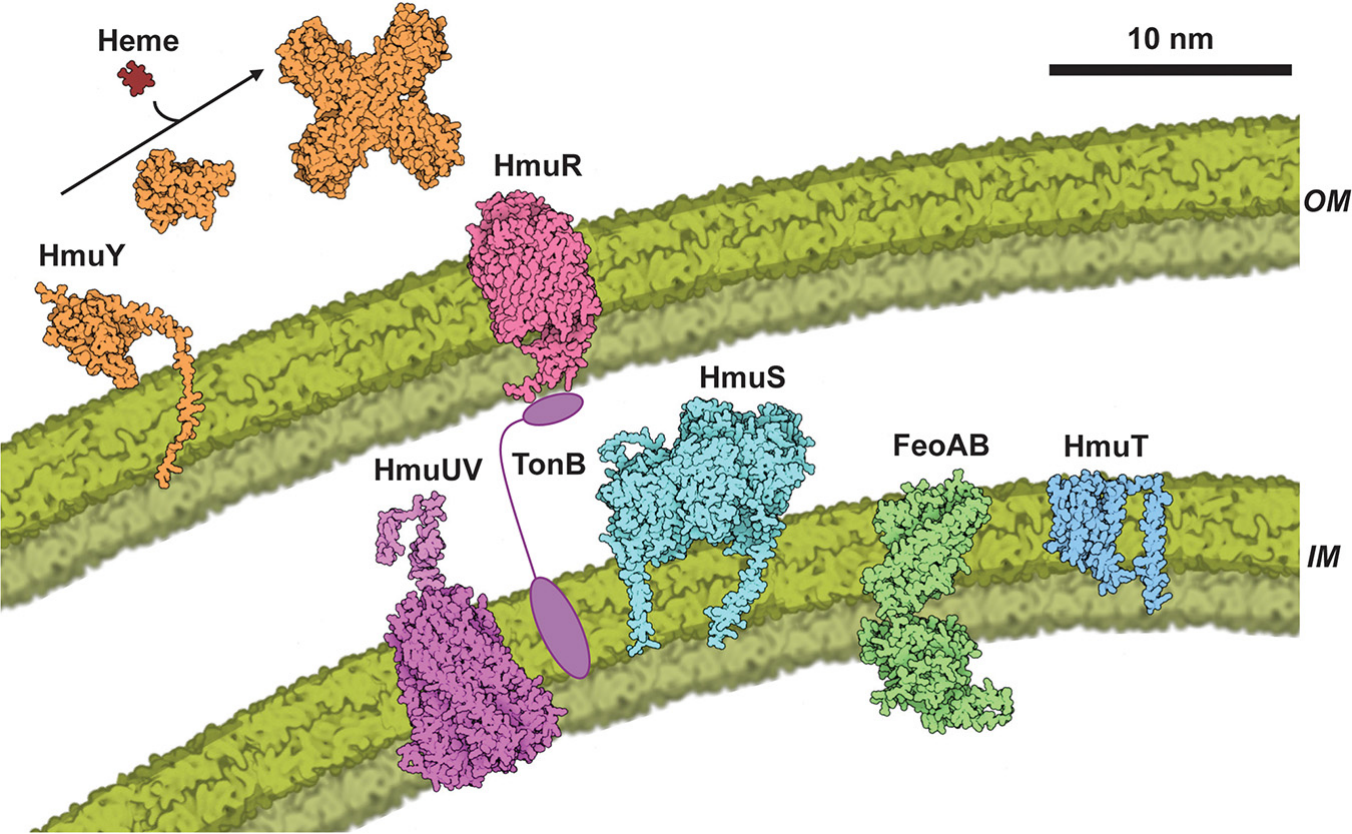
Schematic representation of Hmu proteins involved in heme uptake, transport, and utilization in B. thetaiotaomicron strain VPI 5482. The AlphaFold-predicted structures of B. thetaiotaomicron VPI 5482 proteins from Table S5 were rendered to scale using CellPAINT (64) and arranged to illustrate their postulated roles in the metabolism of heme in *Bacteroidetes*. As described in the text, HmuY (orange) is predicted to be a monomeric, membrane-anchored protein that is proteolytically released into the extracellular milieu, where it binds to heme from an unknown source, forms a homotetramer, and transfers heme to the TonB-dependent receptor HmuR (salmon) at the outer membrane (OM). Heme is transported through the OM and into the periplasmic space via the TonB (purple ovals) and HmuU/HmuV (magenta) proteins, where it may be received by an unknown hemophore. Iron is removed from the heme within the periplasm in a process that requires HmuS, which is anchored into the inner membrane (IM) via two predicted transmembrane helices at its N and C termini. Fe(II) is then transported by FeoAB into the cytoplasm. The function of IM-bound HmuT is not known, but it may act to transport unmetabolized heme into the cytoplasm, thereby meeting cellular demand for heme in fumarate reductase and potentially other proteins.

Two anaerobic mechanisms for iron release from heme have been described or proposed. The reference genome of B. thetaiotaomicron strain VPI 5482 (GenBank accession number NZ_UYXG01000001.1) lacks an operon homologous to the first of these, encoded by the *chu* operon and described for Escherichia coli (2, 36). However, B. thetaiotaomicron appears to possess an *hmu* operon for heme uptake, composed minimally of 6 genes, *hmuYRSTUV* (Fig. 4). Based on the Hmu protein sequences from the reference genome of B. thetaiotaomicron strain VPI 5482 (Table S4), we generated structural models of its Hmu proteins using AlphaFold (37). The protein models were color-coded based on pLDDT values (0 to 100 scale), which is a proxy for local quality of the predicted structure (Table S5). All models except for HmuV have average pLDDT scores between 87 and 90, which indicates that they are very good to excellent models for most of the structure and might be unreliable only in some local details. HmuV likely needs HmuU to fold correctly and some of its parts appear disordered in isolation, consistent with its relatively low score of 70. Overall, the AlphaFold-predicted structures for these proteins broadly support their primary-sequence-based functional assignments (Fig. 4 and Table S5) and are broadly similar to structurally characterized homologs available in the Protein Data Bank.

Homologous operons have been previously identified in Porphyromonas gingivalis and B. fragilis (1, 25). Heme uptake via the Hmu proteins is expected to begin with HmuY, an extracellular hemophore which has a high degree of homology with the HmuY from P. gingivalis (26, 38–40) and Bacteroides vulgatus (Phocaeicola vulgatus) (41). The N-terminal hydrophobic region of B. thetaiotaomicron HmuY is followed by a cleavage site for signal peptidase II (following alanine 23). This suggests that the protein is membrane anchored and released into the extracellular milieu following proteolytic cleavage (26). Sequences involved in oligomerization of heme-bound HmuY monomers to form a tetramer (40) are conserved in the protein from B. thetaiotaomicron. However, while P. gingivalis HmuY binds heme via a *bis*-histidine motif (Fig. S5), the B. thetaiotaomicron protein has a pair of methionine residues at the analogous positions in the primary sequence, similar to the HmuY proteins from the closely related gut commensal Bacteroides vulgatus (41) and the periodontal pathogens Tannerella forsythia (42) and Prevotella intermedia (38, 43). This difference in heme coordination may be responsible for differing affinities of these HmuY proteins for heme.

Homology arguments suggest that the HmuR, -U, and -V proteins are part of a multiprotein system for importing heme through the outer membrane. HmuR has strong sequence and structural similarity to TonB-dependent heme receptors with an N-terminal extension domain (39). Such extensions serve as anti-sigma factor regulatory systems in pathogenic heme uptake pathways (3, 34). In B. thetaiotaomicron, several TonB-dependent receptors with N-terminal extensions, characterized as PF13715 carboxypeptidase D regulatory domains, have been identified. These are proposed to have roles in recognizing nutritional substrates for import into the cell (44). HmuU and HmuV in B. thetaiotaomicron show strong sequence and predicted structural similarities to ExbB and ExbD, respectively. These proteins form a proton channel in the inner membrane which, along with a TonB protein (45) that is not part of the *hmu* operon, couple the outer membrane receptor (HmuR) to the proton motive force in the inner membrane and thereby drive heme import (45, 46).

HmuT in B. thetaiotaomicron lacks structural or sequence homology to other bacterial proteins but possesses a series of predicted transmembrane-spanning regions, suggesting that it is membrane bound. In prior literature, HmuT is variously described as a periplasmic binding protein (47) and annotated as part of the type II class of ABC transporters (48). Based on its predicted membrane localization, the need for both Fe(II) and heme in the B. thetaiotaomicron cytoplasm (49), and the specific association of *hmuT* genes with the *hmu* operon (described below), we speculate that it could play a role in transport of unmetabolized heme through the inner membrane and into the cytoplasm. HmuS, a member of the CobN/CbiX family of metal/porphyrin chelatases, is hypothesized to be necessary for catalyzing removal of ferrous iron from heme in Gram-negative bacteria (1, 4, 50–52). An inner membrane-associated FeoAB protein involved in ferrous iron import to the cytoplasm was identified in a pathogenic strain of B. fragilis (4), suggesting that any iron releasing activity by HmuS and/or other proteins is periplasm directed. Experimentally, it was shown that the single gene knockout of one putative HmuS-encoding gene (*btuS2*) and a *btuS2 btuS1* double knockout in which both paralogous genes are inactivated, exhibited the expected growth defects when the mutant strains were grown on heme as the iron source. Growing the double-knockout mutant on heme, remarkably, led to apparent PPIX accumulation, though the reasons for this phenotype are unclear. Our ongoing work is consequently focusing on structural and biochemical studies of HmuS.

### Operonic composition, synteny patterns, and phylogenetic distribution suggest that the *hmu* operon is widespread within the *Bacteroidetes* phylum, with two genes specific to the operon.

We investigated the distribution of the *hmu* operon in bacterial genomes using *cblaster* (53), with protein sequences from B. thetaiotaomicron strain VPI 5482 (GenBank accession number NZ_UYXG01000001.1) as bait (Table S5). A total or partial *hmu* operon was present in 4,490 bacterial genomes (Data Set S1), the majority of which (~99%) belonged to the phylum *Bacteroidetes* (Table S5). The conservation pattern with all 6 genes being present in the genome (that is, 111111) was most prevalent. A complete *hmuYRSTUV* operon was detected within 2,170 species of the *Bacteroidetes* phylum (Table S5), which includes strains of the Gram-negative bacterial species Bacteroides thetaiotaomicron, Bacteroides fragilis, and Porphyromonas gingivalis; many strains of the latter two species are human pathogens in anaerobic microenvironments. Other conservation patterns were also abundant: 010111, in which HmuY and HmuS were not detected (981 species); 011111, in which HmuY was absent (385 species); 000111 (337 species), lacking HmuY, -R, and -S; and 011011, in which both HmuY and HmuT were absent (140 species). The lack of a homologous HmuY protein indicates one of two possibilities: (i) the HmuY sequence in many species seems to be divergent/specific to different heme complexes, and its identification may escape the 30% identity threshold, and (ii) the *hmuY*-deficient operons are not involved in heme transport, as they do not code for the hemophore HmuY.

**FIG 5:**
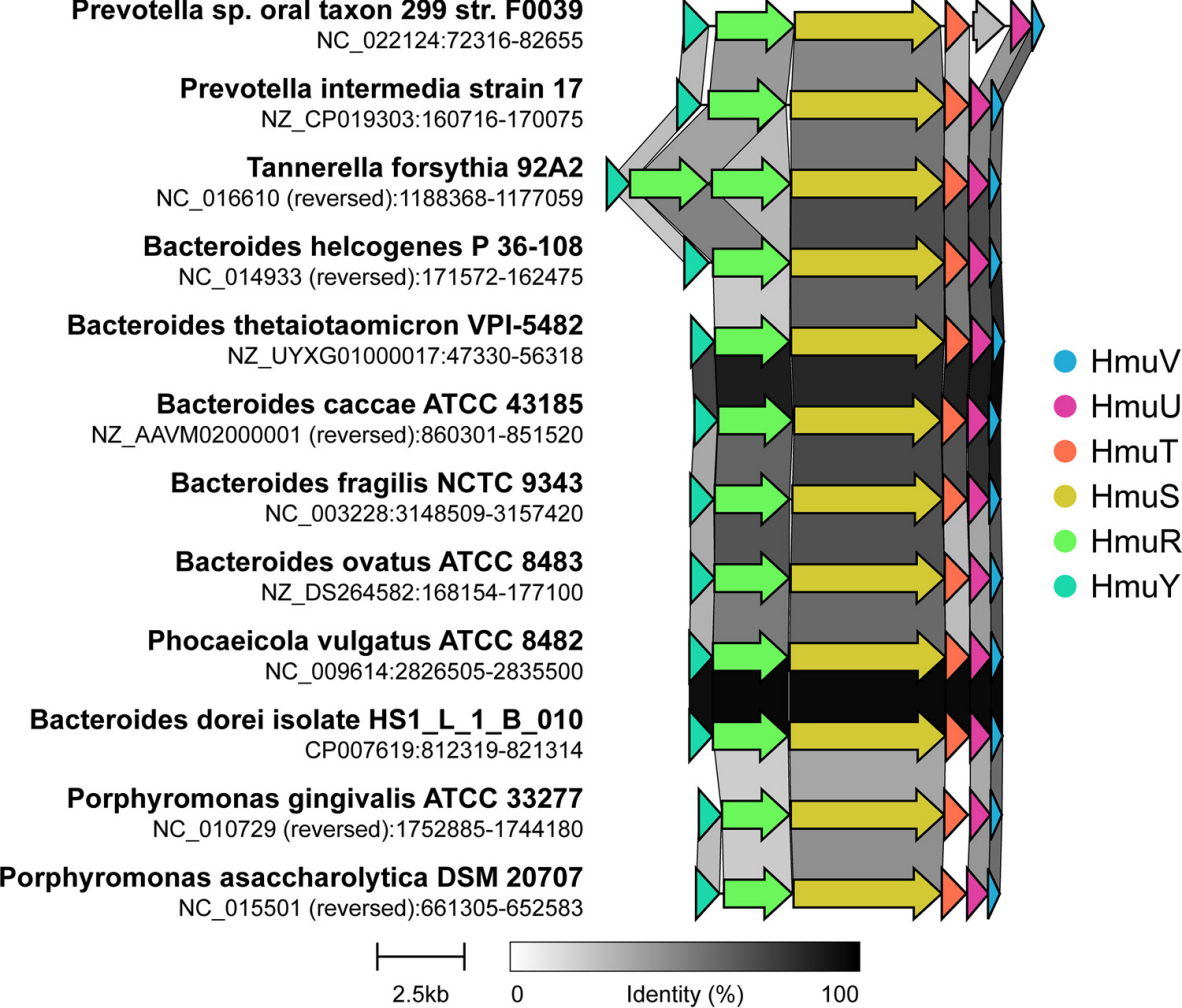
Synteny of the *hmu* is maintained within *Bacteroidetes*, as illustrated by operon diagrams from selected major Gram-negative species. Sequence homology of *hmu* proteins in major gut microbiome and other well-studied species was determined by Clinker (54). The 6 genes composing the *hmu* operon are colocalized in the genomes of illustrated species within the phylum *Bacteroidetes*, from the families *Bacteroidaceae* (*Bacteroides*, *Prevotella*, and *Phocaeicola*), *Porphyromonadaceae* (*Porphyromonas* and *Tannerella*), and *Prevotellaceae* (*Prevotella*).

We used Clinker (54) to globally align the HmuYRSTUV protein sequences from the complete genome of Bacteroides thetaiotaomicron strain VPI 5482 against several major Gram-negative gut microbiome species (15). The results indicate that synteny is maintained within the *hmu* operons of species within members of the phylum *Bacteroidetes* from the family *Porphyromonadaceae* (*Porphyromonas* and *Tannerella*) and *Bacteroidaceae* (*Bacteroides*, *Prevotella*, and *Phocaeicola*) (Fig. 5). Overall, the global function and structure of the *hmu* operon are mostly retained among members of the *Bacteroidetes*, including several common GI tract species and some likely pathogenic strains.

### Signature *hmu* genes are widely distributed among healthy human gut metagenomes.

The distributions of all Hmu proteins in the human gut database and in the general protein database were investigated using custom hidden Markov models (HMMs) (55, 56), to assess which species commonly found in the reference healthy human GI tract are associated with Hmu proteins (Table 3). HmuS, -T, -V, and -Y in the human gut are found almost exclusively in the *Bacteroidetes* phylum, while in a general database some of them have a wider distribution. A complete *hmu* operon, as a group of 6 consecutive genes, is found only in *Bacteroidetes* and is likely to be involved only in heme uptake. There are few exceptions from that rule that have more divergent HmuY and HmuR components. These operons contain a cobalamin riboswitch (see Materials and Methods), indicating that the operon is likely not involved in heme utilization but perhaps is involved in the metabolism of structurally and evolutionarily related cobalamin. For example, in Bacteroides fragilis NCTC 9343, we identified Hmu-like operons that had two consecutive HmuR copies and no HmuY but contained a cobalamin regulatory riboswitch immediately preceding this group of genes (genomic coordinates 2956033 to 2956280).

**TABLE 3:** Hmu proteins are prevalent in *Bacteroidetes* and *Proteobacteria*^*a*^

Protein	Human gut database			UniRef90 database		
	Total hits	Largest clusters	Cluster identifier	Total hits	Largest clusters	Cluster identifier
HmuY	40	9, 6	*Bacteroidetes*	4,873	201, 137	*Bacteroidetes*
HmuR	2,736	36, 33	*Bacteroidetes*, *Gammaproteobacteria*	388,372	1,006, 923	*Bacteroidetes*
HmuS	37	23, 4	*Bacteroidetes*, *Methanomicrobia*	12,302	1,699, 1581	*Bacteroidetes*, *Proteobacteria*, *Cyanobacteria*, *Actinobacteria*, *Chloroflexi*
HmuT	22	8, 5	*Bacteroidetes*	228	37, 29	*Bacteroidetes*
HmuU	204	24, 23	*Bacteroidetes*, *Proteobacteria*	43,574	1,835, 1,487	*Proteobacteria*
HmuV	26	22	*Bacteroidetes*	1,242	233, 229	*Bacteroidetes*, *Proteobacteria*, *Methanomicrobia*

a Distribution of Hmu proteins in the largest clusters of the healthy human gut microbiome database (GutFeeling Knowledgebase) (72) and the general protein database (UniRef90) (73). The occurrence of each protein was calculated using hidden Markov models (55) with a cutoff E value of 1e^−10^.

HmuR is the most represented protein, with 2,388,372 total hits in the UniRef90 database and 2,736 hits in the Human Gut database (Table 3). TonB-dependent outer membrane receptors like HmuR are common in Gram-negative bacterial operons (1, 2, 41, 45). Similarly, ExbB-type inner membrane proteins (HmuU) are widely distributed, with 43,574 and 204 hits against the UniRef90 and the Human Gut databases, respectively (Table 3). HmuY, HmuT, and HmuV, identified as hypothetical proteins in B. thetaiotaomicron strain VPI 5482 (Table S4), have the lowest occurrences in both databases. This may indicate that the hemophore HmuY, the inner membrane-localized HmuT, and the inner membrane transporter/ExbD homolog HmuV are the most specific components of a heme metabolism operon. These three proteins appear to be diagnostic of heme transport and are not used in operons responsible for the transport of other ligands. This contrasts with the widely distributed superfamily of receptors that includes HmuR, where the same fold is used for a variety of ligands.

### Conclusions.

The *Bacteroidetes* are a dominant group of bacteria in many healthy GI tract flora. B. thetaiotaomicron is a commensal heme auxotroph and representative of this group, which we showed preferentially uses and ultimately hyperaccumulates iron in the form of heme, appearing to gain no further growth benefit from the addition of nonheme iron (under the conditions used in this study). By quantifying its iron intake on a per-genome basis, we were able to estimate the amount of iron in a hypothetical microbiome consisting wholly of B. thetaiotaomicron cells. This estimate suggests that the GI tract microbiome could possess an iron store sufficient for replacing several days’ worth of the typical human nutritional iron requirement, constituting a substantial iron reservoir and potential buffer against anemia. A bioinformatics analysis showed that the *hmu* operon, encoding an anaerobic mechanism for heme uptake and metabolism in members of the *Bacteroidetes*, is widespread in but confined to the *Bacteroidetes* phylum. Genes from this operon are likewise widespread in metagenomic samples collected from healthy humans. We consequently expect *Bacteroidetes*- and specifically *hmu*-mediated heme metabolism to constitute a major mechanism for mobilizing iron from host dietary heme on behalf of both the microbiome and host. Such mechanisms must be robust, as heme detected in stool is generally ascribed to host-derived occult blood, where it is diagnostic of bleeding in the lower GI tract (57, 58). Nutritional studies demonstrate that heme is a more bioavailable source of human dietary iron than typical plant-derived nonheme iron complexes; however, even heme iron is incompletely absorbed by the host, demonstrating that it must be shared with the microbiome (32). Though porphyrins are known to be excreted, the various fates of PPIX are unclear.

Preferential heme usage by *Bacteroidetes* may give them a selective advantage in a GI tract environment. Heme promotes robust growth in *Bacteroides* spp. that ferment glucose, due to enhanced conversion of fumarate to succinate by the heme-dependent fumarate reductase (49). Host diets rich in heme-dense red meat are, in turn, associated with a greater proportion of *Bacteroidetes* in the colonic microbiome, relative to members of the other dominant phylum, *Firmicutes* (59, 60). Both *Bacteroides*-dense microbiomes and red-meat diets are associated with the induction of colon cancer (61–63). It is unclear whether these host pathologies relate to the proinflammatory properties of the heme or porphyrins themselves, whether they result from other metabolic activities of the heme-stimulated factions of the microbiome (27), or some combination of the two. In short, rather than promoting pathogenesis outright, preferential accumulation of heme by *Bacteroidetes* in the commensal context may instead lead to species imbalances (dysbiosis) and consequent biochemical disruptions to the host ecosystem.

## MATERIALS AND METHODS

### Growth of B. thetaiotaomicron VPI 5482 on heme and nonheme iron sources.

**(i) Monitoring growth over time.** The anaerobic growth of B. thetaiotaomicron VPI-5482 was assessed via optical density at 600 nm (OD_600_) over time. Cultures were incubated in a Coy anaerobic chamber at 37°C (2.5% H_2_ and 97.5% N_2_ atmosphere) with intermittent shaking between 30-min reads in 24-well Falcon polystyrene non-tissue-culture-treated plates (Corning, NY; 351147) with low binding affinity in volumes of 2 mL media with path length correction. Plates were sealed with sterile, optically clear film (Bio-Rad, CA; MSB1001), and needle pricks were made with a 26-gauge sterile hypodermic needle (Becton, Dickinson and Company, NJ; 10161F) in the film to allow gas exchange without evaporation over 30 h. The OD_600_ was measured every 30 min using a Varioskan LUX 3020-864 plate reader (Thermo Scientific) and 800TS microplate reader (Biotek Instruments), and data were analyzed using ScanIt software 5.0 (Thermo Scientific) and Gen5 version 3.08.01 (Biotek Instruments). Scaled-up anaerobic cultures were grown in Hungate tubes at 37°C under genus-optimal headspace conditions (5% CO_2_, 10% H_2_, and 85% N_2_ atmosphere) (15, 16). OD_600_ was measured using a GENESYS 30 spectrophotometer adapted to Hungate tubes.

The growth curves in Fig. 2 were fitted to the modified Gompertz equation described by Riet (18), using Kaleidagraph: OD_600_(*t*) = *A* exp{−exp[*k*(λ − *t*) + 1]}, and *k* = (μ_m_ *e A*^−1^). The 3 phases of the curve are described by μ_m_, the maximum specific growth rate defined as the slope of the tangent line at the curve’s inflection point (here, in au/time), the lag time λ (the *x*-intercept of this tangent line), and *A*, the asymptote, which is the maximum level of growth (*A* = ln *N*_∞_/*N*_0_).

**(ii) Defined minimal medium for B. thetaiotaomicron: heme and nonheme iron.** To evaluate growth-promoting versus growth-limiting concentrations of heme and nonheme iron in cultures of B. thetaiotaomicron VPI-5482, a defined minimal fermentation medium (MM) adapted from published recipes (15–17) was prepared in plastic Nalgene flasks (Thermo Fisher Scientific, MA) containing 6.6 mM potassium dihydrogen phosphate, 15.4 mM sodium chloride, 98 μM magnesium(II) chloride hexahydrate, 176.5 μM calcium(II) chloride dehydrate, 4.2 μM cobalt(II) chloride hexahydrate, 50.5 μM manganese(II) chloride tetrahydrate, 9.3 mM ammonium chloride, 1.75 mM sodium sulfate, 134 μM l-methionine, 23.8 mM sodium bicarbonate, 8.25 mM l-cysteine (free base), and 28 mM d-glucose into chelated and filtered (0.2 μm) Milli-Q water. The water used to make minimal medium and chemical solutions was chelated with Chelex 100-Na form (Sigma Life Science, MO) in batch at 2 g/L for >1 h before use according to the manufacturer’s protocol to remove any trace iron.

Hemin chloride (Calbiochem, CA; CAS 3741) stock solutions were prepared aerobically at 0.01 M in basic water (1 N NaOH; Fisher) or dimethyl sulfoxide (DMSO; Fisher), and iron(II) sulfate heptahydrate (ARCOS, OH; CAS7782-63-0) stock solutions were prepared anaerobically at 0.01 M in neutral water. Heme and Fe(II) were then added to 1× MM as described in Results. Anaerobic growth in the glove box at 37°C was assessed over time by OD_600_ in a 24-well plate with a 2-mL final volume in each well. These were seeded from an inoculum grown in beef broth for 48 h at 37°C, starting from a B. thetaiotaomicron colony freezer stock scraped with a sterile inoculating loop and transferred into 10 mL fresh beef broth. The inoculum cells were pelleted and washed with 1× MM, then resuspended in 1× MM with the designated heme/iron concentration, and diluted 1:10 into each well in triplicate. Iron supplements were added from stocks immediately prior to use, and total iron concentrations of amended media were verified by AA. A blank well for each condition with 2 mL noninoculated medium was also measured.

**(iii) Correlation of OD measurements to other growth proxies.** Measurements of DNA and wet pellet mass per mL of culture were used as surrogates for liquid or plate counts of CFU (see supplemental material). Cell pellets were collected from 1-mL saturated cultures in triplicate by gentle centrifugation (6,000 rpm for 3 min) and weighed on an analytical balance. DNA was extracted from the wet cell pellets using the Fast DNA spin kit for soil (MP Biomedicals, Irvine, CA), following the manufacturer’s protocol. Total double-stranded DNA (dsDNA) was quantified using a Nanodrop UV/visible spectrophotometer (Thermo Fisher Scientific). The DNA concentration was reported as micrograms per milliliter of B. thetaiotaomicron culture at the given OD_600_. Mean values from triplicate measurements of OD_600_, wet pellet weight, and DNA concentration (Table S1) were used in generating correlation plots shown in Fig. S1. Resulting conversion factors are reported in Table S3.

### Targeted quantification of B. thetaiotaomicron VPI 5482 metabolites.

**(i) Extraction of heme and porphyrin from cell pellets.** Heme and nonheme iron from cell pellets of B. thetaiotaomicron cultures were quantified (Fig. S3). Cells were grown to stationary phase (OD_600_, 1.0 to 1.5) in rich medium (beef broth) or defined medium (minimal medium with hemin with or without FeSO_4_). Triplicate cultures (10 mL each) were pooled in a 50-mL Falcon tube. A subsample of the pool was used for determination of cell pellet weight (milligram per milliliter) and corresponding DNA yield (μg/mL) for normalization of analytical measurements (Table S2). A volume of 20 mL of pooled cultures was used for heme and iron extraction. Cells were pelleted by centrifugation (10,000 rpm for 3 min) and immediately frozen at −80°C until extraction. Spent media were collected upon separation and frozen. The cells were resuspended in 1 mL 1 M HCl-DMSO (1:1 [vol/vol]), lysed using lysing matrix B vials (MP Biomedicals) and a FastPep-24 5G instrument (E. coli setting, 6.0 m/s, 40 s total, twice), and centrifuged (10,000 rpm, 5 min, 4°C), and supernatant was collected in a 15-mL centrifuge tube. The pellet was resuspended and sedimented again in the same way to be certain of complete extraction of iron and porphyrins from cellular debris. The supernatants were pooled (ca. 2 mL total) and equally subsampled for total iron analysis (1.0 mL) and heme iron analysis (1.0 mL) following the protocols described below. The same protocol was also applied to the spent media of the cultures at the end of the incubation, after lyophilization of a 2-mL subsample volume overnight for each analytical method.

**(ii) Total iron measurements via AA.** The 1.0-mL subsample of supernatant from B. thetaiotaomicron cultures was diluted 2× to 6× in chelated Milli-Q water, depending on the growth conditions and expected cellular concentration. Samples were analyzed for total iron by atomic absorption spectroscopy (AA; Agilent 200 series) with flame generated from acetylene and compressed air, and using AA standards (Ricca; 100 ppm; CAS AFE1KH-100) diluted from 0.1 to 4 ppm in chelated deionized water (Fig. S4). Linear regression analysis (Kaleidagraph) was used to determine the correlation coefficient between absorbance and iron concentration (slope of standard curve, *m*_iron_). Absorbance values were converted to units of concentration (micromoles per liter) through the following equation: [*A*] = absorbance × (*m*_iron_)^−1^. The concentration of iron in each sample (nanomoles per milliliter of culture) was then calculated: [*A*] × (dilution factor) × (volume of culture)^−1^. Reported values are averages of technical replicates. The total iron concentration was then normalized to the wet pellet weight and the DNA mass, each a surrogate for CFU counts.

**(iii) Porphyrin analysis via liquid chromatography-mass spectrometry (LC-MS).** The 1.0-mL subsample of supernatant from B. thetaiotaomicron cultures was diluted to 25 mL with chelated distilled water (dH_2_O). On a vacuum manifold, one Sep-Pak Vac 3 cc tC18 cartridge (Waters; 036815) per sample was equilibrated with acetonitrile plus 0.1% trifluoroacetic acid (TFA) and then 4 mL chelated Milli-Q H_2_O and supernatants were added to the column, followed by a wash with 10 mL chelated Milli-Q H_2_O. Sample columns were eluted with 2 mL acetonitrile plus 0.1% TFA followed by 2 mL methanol, evaporated using a SpeedVac (V-AL vent mode; Vacufuge Plus; Eppendorf) at 45°C for >6 h, and concentrated porphyrins were stored protected from light at −80°C until quantification.

Porphyrin concentrates were resuspended in 100 to 1,000 μL acetonitrile plus 0.1% TFA depending on the growth conditions and expected cellular concentration. A volume of 100 μL was transferred to a small-volume tapered MS sample vial for analysis. To generate a standard curve for absolute quantification, a mixture of 2 porphyrin standards (2 mM) was prepared in DMSO and diluted to 100 μM hemin chloride (651.94 g/mol) and protoporphyrin IX (molecular weight [MW], 562.26 g/mol) in acetonitrile plus 0.1% TFA. This stock was serially diluted (0.25 to 6 μM) in acetonitrile plus 0.1% TFA and analyzed by LC-quadrupole time of flight (Q-TOF) MS in positive-ion mode (Fig. S4). The mass per charge [*m/z*] value and elution time for each compound are reported in Fig. S4.

Samples were analyzed on an Agilent 6538 ultrahigh-definition (UHD) Q-TOF instrument using a PLRP 5-cm column (Agilent) equilibrated to an 85:15 ratio of solvent A (ultrapure water with 0.1% formic acid) to solvent B (acetonitrile plus 0.1% formic acid). Liquid chromatography separations were achieved by linear gradient elution, transitioning from 15% to 95% solvent B over 6 min followed by a 2-min hold at 95% solvent B. The column was reequilibrated to 15% solvent B for 2 min in between injections of the same sample (two technical replicates run per sample, 2-μL injection volume, 600-μL/min flow rate, 50°C). Two blank samples were run between samples to ensure against column holdover of analytes. Electrospray ionization-mass spectrometry analysis was carried out in positive mode with a capillary voltage of 2 Hz.

Values for *m/z* were determined empirically for standards containing heme and PPIX. From the total ion chromatogram (TIC) traces (Fig. 3A), extracted ion chromatograms (EICs) were integrated for each individual standard based on its *m/z* in positive-ion mode (Fig. 3B), which is equivalent to the exact mass of its positive ion (parent compound plus H^+^) using Mass Hunter Qualitative Analysis software version 10.0. Peaks associated with each analyte were integrated and areas were plotted versus concentration (micromolar). Linear regression analysis (Kaleidagraph) was used to determine the correlation coefficient between integrated peak area and porphyrin concentration (slope of standard curve [m_porphyrin_]) where porphyrin corresponds to analyte of interest. All cell samples were analyzed for both standards. Only analytes present above the limit of detection are reported; detection limits were at least 250 nM per injection for a saturated culture. For the quantification of analytes from cells, values for the integrated peak intensities (measured in units of ion counts) were converted to units of concentration (micromoles per liter) through the following equation: [*A*] = counts × (*m*_porphyrin_)^−1^. The concentration of each analyte in the sample (nanomoles per milliliter of culture) was then calculated as follows: [*A*] × dilution factor × (volume of culture)^−1^. Reported values are averages of technical replicates. The heme and PPIX concentration were then normalized to the wet pellet weight and the DNA mass, each a surrogate for CFU counts.

### Bioinformatics analysis of the composition and distribution of a likely operon encoding heme uptake and metabolism.

**(i) Structure and function of the *hmu* operon based on protein sequence.** B. thetaiotaomicron appears to possess an *hmu* operon for heme uptake, composed of 6 genes (*hmuYRSTUV*) transcriptionally regulated by the iron-binding transcription factor Fur. Homologous operons have been previously identified in P. gingivalis (1, 26) and B. fragilis. The protein sequence of each operonic unit (Table S4) was used to predict protein function with the NCBI database (https://www.ncbi.nlm.nih.gov/protein) and protein structure with the AlphaFold model (https://alphafold.ebi.ac.uk/). AlphaFold calculates a per-residue confidence measure called pLDDT (0 to 100 scale), and this local measure of model quality is stored in the B-factor column of the Protein Data Bank (PDB) (37). Coloring was done by B-factor in PyMol (https://pymol.org/2/) (64): highly accurate model regions are shown in red, orange, and green and correspond to confidently modeled regions, while blue regions are modeled with low confidence (Table S4). The latter model parts are generally not informative except possibly as disordered regions. NCBI BLAST was then used to search for structurally characterized homologs based on amino acid sequence. Sequence alignments were made with Clustal Omega (65) and viewed with Jalview (66). Protein topology was determined using DeepTMHMM (67) and SignalP (68). AlphaFold structures were aligned to known crystal structures of homologs using SwissModel (69) and PyMOL (64). The schematic representation of the Hmu proteins in Fig. 4 was constructed with proteins plotted to the same relative length scale using Illustrate (70) and CellPAINT (71).

**(ii) Hmu operon homology and synteny in bacterial genomes.** Protein sequence homology in the *hmu* operon was investigated across bacterial genomes using Clinker (54) to globally align the HmuYRSTUV protein sequences from the complete genome of Bacteroides thetaiotaomicron strain VPI 5482 (GenBank accession number NZ_UYXG01000001.1) against major Gram-negative gut microbiome species. Hmu protein homology was defined as ≥30% sequence identity, and the conservation pattern of the 6 genes of the *hmu* operon was expressed in a binary table (Table S5) using *cblaster*, with scoring as follows: absence, 0; presence, 1; and two copies, 2 (53). Operon synteny was evidenced by the colocalization of all 6 genes in species within the phylum *Bacteroidetes* (Fig. 5), from the families *Bacteroideaceae* (*Bacteroides*, *Prevotella*, and *Phocaeicola*), *Porphyromonadaceae* (*Porphyromonas* and *Tannerella*), and *Prevotellaceae* (*Prevotella*). Cobalamin riboswitch annotations could be identified preceding some gene clusters that resembled *hmu* operons but lacked HmuY or other components. Such riboswitches are identified in most genomic annotations that have been created in the past 10 years, like most of those examined in this study. For older genomes that may lack cobalamin riboswitch annotation, they could be identified in a straightforward manner using the RF00174 model from Rfam (https://rfam.org/family/RF00174) and “cmsearch” software from the Infernal suite (http://eddylab.org/infernal/).

**(iii) Distribution of Hmu proteins in the healthy human gut microbiome.** The phylogenetic distribution of the Hmu proteins in the healthy human gut was investigated using HMMs (55). Homologs for proteins of interest were collected and aligned automatically after 5 search iterations with HHblits (56), and their alignments were converted to HMMs suitable for the HMMer 3 suite of programs (55). Search parameters were set to identify even divergent relatives, as we were interested in global distributions of all proteins with similar functionality. The occurrences of the individual proteins in the GutFeeling Knowledgebase reference database (https://hive.biochemistry.gwu.edu/gfkb) of healthy human gut microbiome (32) were summarized (Table 3). The occurrence of each protein in the UniRef90 database (clustering sequences at 90% sequence identity) was also investigated (16).
