# Supplementary material for: Bacteroides thetaiotaomicron, a Model Gastrointestinal Tract Species, Prefers Heme as an Iron Source, Yields Protoporphyrin IX as a Product, and Acts as a Heme Reservoir
Source: Microbiol Spectr. 2023 Mar 2;11(2):e04815-22. doi: 10.1128/spectrum.04815-22 (PMC10100974; doi:10.1128/spectrum.04815-22)
Supplement: Supplemental file 1 — Supplemental text, Fig. S1 to S5, and Tables S1 to S5. Download spectrum.04815-22-s0001.pdf, PDF file, 4.5 MB [file spectrum.04815-22-s0001.pdf]

SUPPLEMENTARY INFORMATION FOR THE MANUSCRIPT:

Bacteroides thetaiotaomicron, a model gastrointestinal tract species, prefers heme as an iron source, yields protoporphyrin IX as a product, and acts as a heme reservoir

Margaux M. Meslé[1], Chase R. Gray[1]Mensur Dlakić[2], and Jennifer L. DuBois[1*]

[1]Department of Chemistry and Biochemistry, Montana State University, Bozeman, MT 59717, USA

[2]Department of Microbiology and Cell Biology, Montana State University, Bozeman, MT 59717, USA

*Corresponding author: DuBois, Jennifer L. (jennifer.dubois1@montana.edu)

This PDF file includes:

Supplementary methods and results

Figures S1 to S5

Tables S1 to S5

SI literature references

SUPPLEMENTARY METHODS AND RESULTS

Selected strain and isolation in rich media. A non-pathogenic strain of *Bacteroides thetaiotaomicron* was purchased from the American Type Culture Collection (ATCC). This strain, ATCC 29148™, corresponds to the strain VPI-5482 for which the genome has been fully sequenced (1). The freeze-dried stock cells were resuspended anaerobically in a glove box into 6 mL of a modified Chopped Meat medium (ATCC 1490) prepared according to ATCC methods (2, 3). The heme content of the beef broth was below the detection limit of the pyridine hemochromagen assay at (<1 μM) (4, 5). The cells were then transferred to a sterile Hungate tube with a 5% CO_2:H_2 N_2 headspace and incubated at 37°C for 30 hours. A 150 μL aliquot of the culture was spread onto a Tryptic Soy Agar (TSA) + 5% sheep blood plate (ATCC 260 medium) prepared according to ATCC methods (2). Additional agar plates consisting of Brain Heart Infusion medium supplemented (BHIS) with hemin and vitamin K1 (2, 3), Luria Bertani (LB Miller) medium supplemented (LBS) with hemin, $FeSO_4$ and vitamin K1, and Chopped Meat medium with agar, were used in parallel to isolate single *Bt* colonies (Figure S1). Colony morphologies resembled small (< 3 mm), white, waxy circles. However, numbers of colonies did not correlate well with volumes of liquid culture (from cultures having equivalent optical densities). We hypothesized that cell clumping, especially in the presence of hemin, could be responsible for the observed poor correlations.

Identification of isolated colonies by PCR and microscopy. To confirm the identity of the colonies isolated from different agar media (Figure S1), the primer pair Bfr-F (5’-CTGAACCAGCCAAGTAGCG-3’) and Bfr-R (5’-

CCGCAAACTTTCACAACTGACTTA-3') targeting the 16S region of species of the *B. fragilis* group, as well as the primer pair 1392A (5'-GTACACACCGCCCGT-3') and Bth-R (5'-ACCTATGAAATCGTTGTTACG-3') targeting the 16S-23S rRNA intergenic spacer region of *B. thetaiotaomicron* species, were used to amplify Bacteroides or *Bt*-specific DNA sequences (6). The 20 µL PCR reactions included 10 µL of 2X Dream Taq Master Mix, 1 µL of each 10 µM primer stock, nuclease-free water, and 1 µL of colony DNA template. The negative control consisted of a reaction using nuclease-free water in place of the DNA template. PCR was carried out for 35 cycles: 95°C for 20 s for denaturation, annealing for 1 min at 52°C (Bfr-F/Bfr-R) or 62°C (1392A/Bth-R), extension at 72°C for 30 s. A cycle of 72°C for 5 min was added for the final extension. PCR products were analyzed by electrophoresis on a 1% agarose gel and imaged with a trans illuminator after Sybr Safe staining. Both sets of primers showed positive amplification from the different colonies, confirming that *Bt* was isolated and growing on the different media (Figure S1).

Field Emission Scanning Electron Microscopy (FE-SEM) images of representative *Bt* cultures (Figure S1) revealed 1 µm-long rods clumping together. Cells grown to stationary phase in rich medium were fixed in 2.5% glutaraldehyde (Electron Microscopy Science), and a 5 µL sample was mounted and coated with iridium for imaging (1kV) using a Supra 55 field emission scanning electron microscope (Zeiss), housed in the Image and Chemical Analysis Laboratory (ICAL) of Montana State University.

Monitoring growth by optical density. The anaerobic growth of *B. thetaiotaomicron* VPI-5482 was assessed via optical density readings at 600 nm (OD_{600})

over time. Incubations in a Coy anaerobic chamber at 37°C (2.5% H_2 and 97.5% N_2 atmosphere) occurred with intermittent shaking between 30-minute reads in 24-well FALCON polystyrene non-tissue culture treated plates (Corning, New York; 351147) with low binding affinity in volumes of 2 mL of media with path length correction. Plates were sealed with sterile, optically clear film (BIO-RAD, California; MSB1001) and needle pricks were made with a 26G sterile hypodermic needle (Becton, Dickinson and Company, New Jersey; 10161F) in film to allow gas exchange without evaporation over 30 hours. The OD 600 nm was measured every 30 minutes using a Varioscan LUX 3020-864 plate reader (Thermo Scientific) and 800TS microplate reader (Biotek Instruments) and data were analysed using ScanIt Software 5.0 (Thermo Scientific) and Gen5 version 3.08.01 (Biotek Instruments). Scaled-up anaerobic cultures were performed in Hungate tubes and incubated at 37°C under genus-optimal headspace conditions (5% CO_2, 10% H_2 and 85% N_2 atmosphere) (2, 3). OD_{600} was measured using the GENESYS 30 spectrophotometer adapted to Hungate tubes.

Correlation of OD measurements to other growth proxies. Because of microscopically observed cell clumping, which could influence both liquid cell counting and counting of colonies on solid plate media, we made measurements of DNA and wet pellet mass per mL of culture as surrogates for plate counts of colony forming units (CFU). Cell pellets were collected from 1 mL saturated cultures in triplicate by gentle centrifugation (6,000 rpm for 3 min) and weighed on an analytical balance. DNA was then extracted from these wet cell pellets using the Fast DNA Spin Kit for Soil (MP Biomedicals, Irvine, CA), following the manufacturer's protocol. Total dsDNA was quantified using a Nanodrop UV/visible spectrophotometer (ThermoFisher Scientific).

The DNA concentration was expressed in μg/mL of *B. thetaiotaomicron* culture. Mean values from triplicate measurements of OD_{600}, wet pellet weight and DNA concentration (Table S2) were used to generate correlation plots shown in Figure S2 and the conversion factors computed from the slopes of those plots in Table S3.

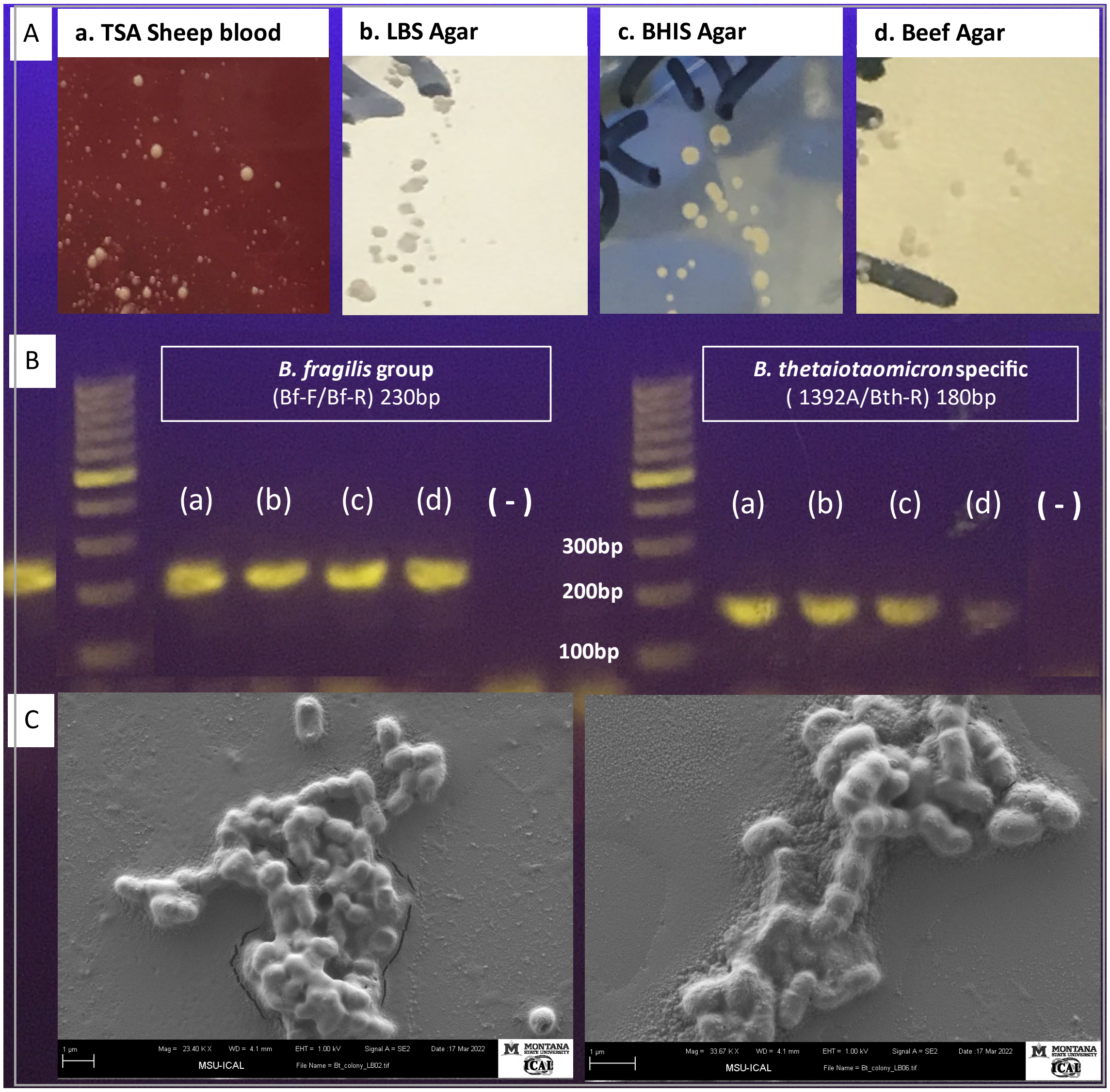

Figure S1. Confirmation of the identity of *Bacteroides thetaiotaomicron* grown on solid and liquid media. (A) Colonies from the ATCC 29148 stock were isolated on several agar media. All colonies were identified as *B. thetaoiotaomicron* and appeared light yellow to orange in color with a smooth texture. (B) Two sets of primers were used to perform PCR amplification (6), one targeting the *B. fragilis* group (16S), and another targeting specifically *B. thetaiotaomicron* (16S-23S rRNA intergenic spacer region). The amplicons were revealed by UV on a 1% agarose gel

after electrophoresis, and their size was verified using the 100 bp DNA ladder. TSA: Tryptic Soy Agar, LBS: Luria Bertani Supplemented, BHIS: Brain Heart Infusion Supplemented (2). (-) Negative controls: *E. coli* colony DNA. (C) FE-SEM images of *Bt* cells which appear as 1 μm-long rods clumped together in a RM culture. (See the legend, in which the length bar represents one micron.)

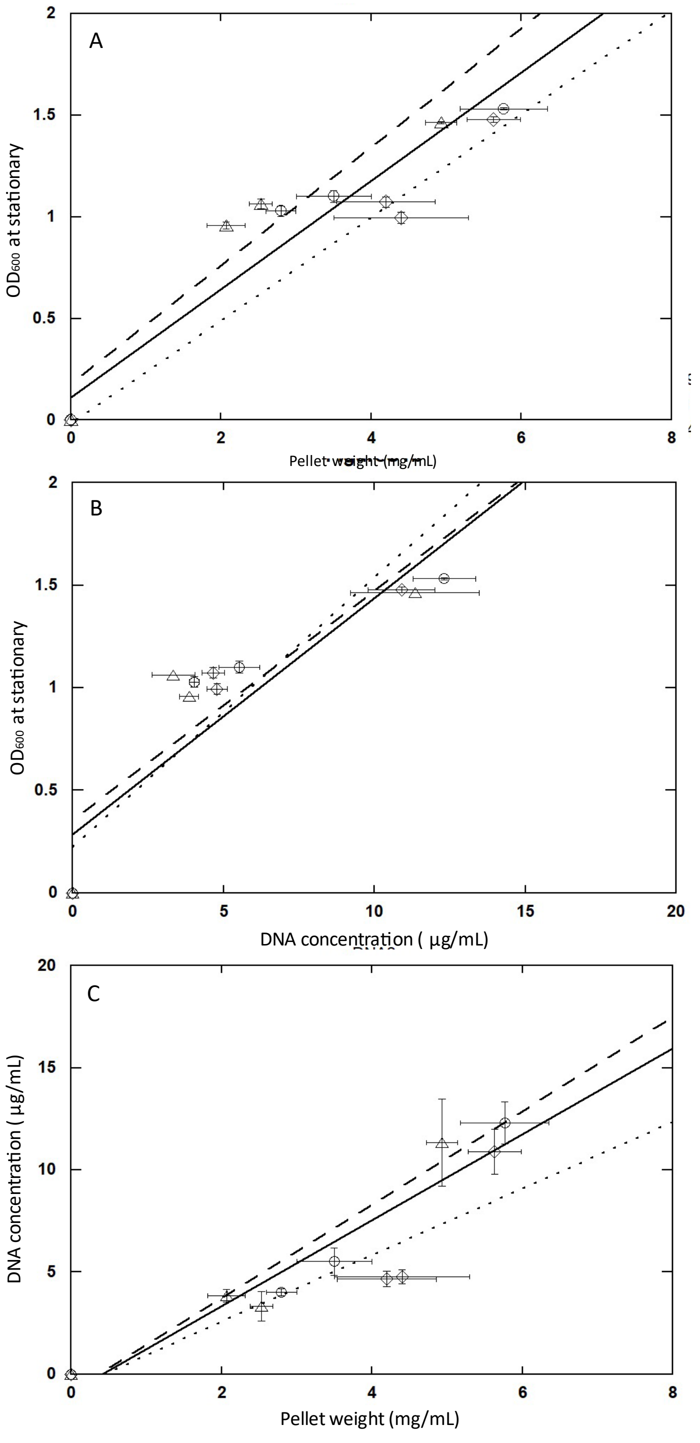

Figure S2. Correlation of *Bt* growth measured optically to pellet mass/volume and DNA concentration. Cultures were grown in rich medium (RM) or minimal medium (MM) supplemented with hemin ± non-heme iron. The different symbols (circle, diamond, triangle)

represent measurements from 3 biological replicates, and the error bars represent ±1 standard deviation from 3 technical replicates obtained for each averaged data point. Lines were fit to each data from each biological replicate, and slopes and intercepts are recorded in Table S3. The plots include 3 major growth proxies: OD_{600} readings at stationary phase, which was correlated with (A) measurements of wet cell pellet mass per 1 mL culture, and (B) corresponding DNA mass obtained per 1 mL of culture. The correlation between DNA and cell pellet masses is shown in (C).

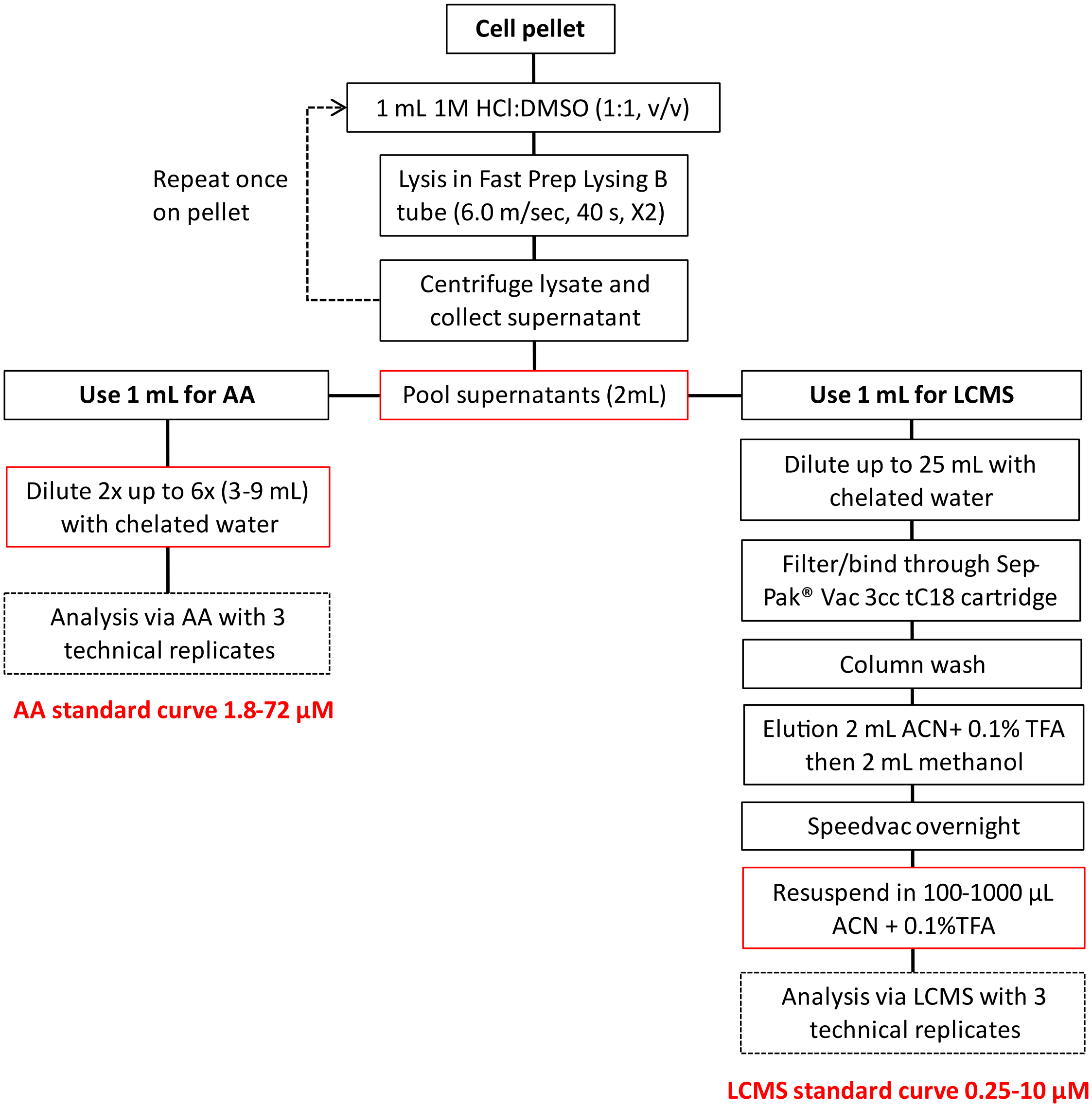

Figure S3. Schematic summarizing extraction and fractionation protocol used to generate analytical samples of *Bt* cells for analysis of total iron and porphyrins (heme and PPIX) contents. This protocol aims at combining early steps of cell preparation by extracting iron and porphyrins from culture pellets for downstream measurements of total intracellular iron as well as intracellular heme and PPIX via AA and LCMS, respectively. The concentration range of the standard curve used for each method is indicated. ACN: acetonitrile.

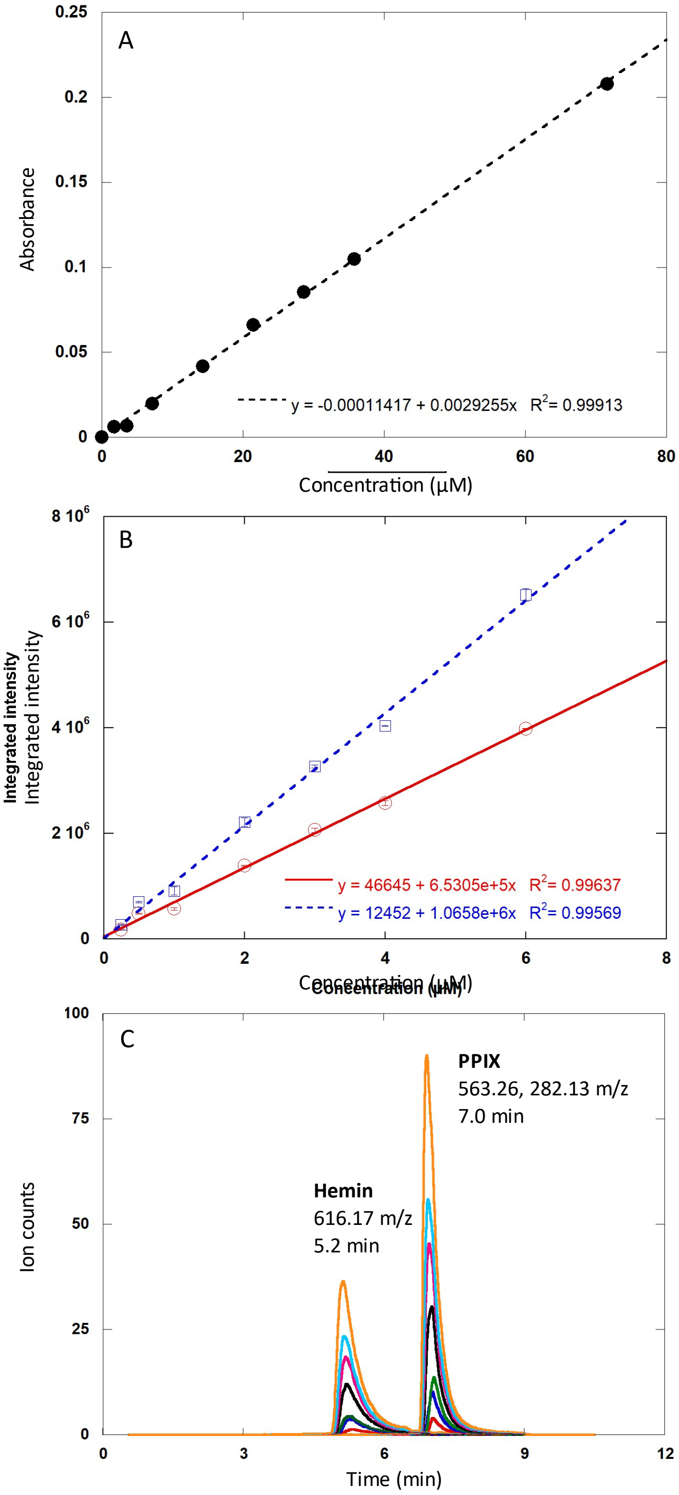

Figure S4. Standards used for total iron, heme and PPIX quantification. (A) Standard curve for AA quantification of total iron ranging from 0 to 4 ppm (0 to 72 µM). (B**)** Standard curves for

LCMS quantification of hemin (red) and PPIX (blue), with error bars representing 3 technical replicates. (C) Corresponding EICs of all LCMS standards. The molecular weight, m/z value, and elution time for each compound is reported. Heme was detected as 616.17 m/z and PPIX as the sum of the ionisation points 563.26 m/z and 282.13 m/z. Linear regression analysis was used to determine the correlation coefficient between measured value and and compound concentration (slope of standard curve, $m_{compound}$).

```
Bt_HmuY   140 GMMEGNIKYTEDYRNDILSGWLNVDTSSMPPIYT 173
Bv_Bvu    106 GMMDGNIVYMESYYNEELSKWLNVDKSNMPPTYT 139
Bf_Bfr    111 HM--GYLIYAPSDFNPELSXWLNVDTSEMPPIYT 142
Pi_PinO   116 RMQSEGVLGMVKTMLNGEMGKWVK-SNGMGKPKT 148
Tf_Tfo    118 DMMKSKFTVAGMAKVNPVLKTWIV-ENPMGKAPV 150
Pg_HmuY   108 GHQMEYEEQGFSEVITGKKNAQGFASGGWLEFSH 141
```

Figure S5. Protein sequence alignments of HmuY from *Bt* VPI-5482 (top) against homologs from selected gut Bacteria. Sequences were chosen from structurally characterized HmuY proteins identified by homology searches against the Protein Data Bank. Alignments were performed using ClustalΩ, and non-Bacteroides alignments were adjusted manually using Jalview to align heme ligating residues (methionine, highlighted in cyan and histidine in red). *Bv: Bacteroides vulgatus, Bf: Bacteroides fragilis, Pi: Prevotella intermedia, Tf: Tanerella forsythia, Sd: Shigella dysenteriae, Sm: Serratia marcescens.*

Table S1. Calculated growth rate and generation time of *Bt* cells grown in optimal to sub-optimal concentrations of iron. Data from Figure 2 were fit to the Gompertz equation: $OD_{600}(t) = A \exp\{-\exp[k(\lambda-t)+1]\}$, where $k = (\mu_m e A^{-1})$, μ_m is the maximum specific growth rate, defined as the slope of the tangent line at the growth curve's inflection point (here, expressed in au/time), λ is the lag time in hours (the x-intercept of this tangent line), and A is the saturation point ($A = \ln N_\infty/N_0$) where $N_\infty = OD_{600}$ at $t = \infty$, $N_0 = OD_{600}$ at $t = 0$.

**growth condition**	**saturation**	**growth rate**		**lag time**
	$\mathbf{A = \ln N_\infty/N_0}$	μ_m **(au/h)**	**(cells/h)**	λ **(h)**
Rich medium 1	1.49	0.232		4.25
Rich medium 2	1.41	0.218		3.69
Rich medium 3	1.47	0.330		6.86
Average	1.46 ± 0.043	0.260 ± 0.061	2.34 (±0.55) x 10^8	4.94 ± 0.17
MM‡ + hemin 1	1.05	0.101		13.4
MM + hemin 2	1.20	0.100		17.6
MM + hemin 3	1.08	0.078		15.3
Average	1.11 ± 0.08	0.0930 ± 0.013	0.840 (± 0.12) x 10^8	15.4 ± 2.0
MM + Fe + hemin 1	1.14	0.103		14.6
MM + Fe + hemin 2	1.24	0.104		16.1
MM + Fe + hemin 3	1.13	0.0996		14.2
Average	1.17 ± 0.062	0.102 ± 0.0023	0.919 (± 0.21) x 10^8	15.0 ± 1.0
MM (no Fe)	0.475	0.00846	0.0761 x 10^8	9.64

‡MM = minimal medium

Table S2. *Bt* cell growth measurements at stationary phase in optimal to sub-optimal conditions of iron. Growth measurements of *B. thetaiotaomicron* strain VPI-5482 cultured in rich medium (RM) and defined minimal medium (MM) supplemented with 15 µM of hemin (Heme) in the presence or absence of 15 µM of non-heme iron ($FeSO_4$, Fe). (A, B, C) Biological replicates of *Bt* cultures grown under different media conditions concentrations. (D) *B*t growth without added iron, and initial data for the inoculum of each biological replicate. The cultures were incubated at 37°C under 10% H_2 + 5% CO_2 in the headspace. Optical density at 600 nm (OD_{600}), wet pellet mass and corresponding extracted DNA mass were all used as proxy for cell growth. All values are the mean ± SD of 3 culture tubes per growth condition.

**A** Replicate 1	**RM**	**MM + Heme + Fe**	**MM + Heme**
Final OD_{600}	1.53 ± 0.01	1.10 ± 0.03	1.03 ± 0.03
Wet PM (mg)	5.77 ± 0.59	5.00 ± 0.50	2.80 ± 0.20
DNA (µg)	12.31 ± 1.01	5.52 ± 0.67	4.03 ± 0.17

**B** Replicate 2	**RM**	**MM + Heme + Fe**	**MM + Heme**
Final OD_{600}	1.48 ± 0.01	1.07 ± 0.03	0.99 ± 0.03
Wet PM (mg)	5.63 ± 0.35	4.20 ± 0.66	5.40 ± 0.90
DNA (µg)	10.89 ± 1.10	4.66 ± 0.38	4.78 ± 0.34

**C** Replicate 3	**RM**	**MM + Heme + Fe**	**MM + Heme**
Final OD_{600}	1.46 ± 0.00	1.06 ± 0.02	0.96 ± 0.02
Wet PM (mg)	4.93 ± 0.21	2.53 ± 0.15	2.07 ± 0.25
DNA (µg)	11.34 ± 2.13	3.33 ± 0.71	3.85 ± 0.32

**D** Controls	**MM No Fe**	**Inoculum 1**	**Inoculum 2**	**Inoculum 3**
Final OD_{600}	0.37 ± 0.02	1.57	1.49	1.55
Wet PM (mg)	1.87 ± 0.31	6.89	6.50	6.70
DNA (µg/mL)	0.91 ± 0.07	24.65	21.16	23.82

Table S3. Conversion factors correlating optical density, pellet mass, and DNA mass, from linear equations fitted to the data shown in Figure S2. Cells were collected as cultures reached saturation, as shown in Figure 2. For each correlation plot, the slope, intercept and R^2 value of the linear curves are indicated. The conversion factor was calculated using average values and the linear regression equation *x=(y-intercept)/slope.* OD: optical density at 600 nm. PM: cell pellet mass in mg, obtained in each case from 1 mL culture. DNA: quantity in µg, obtained from 1 mL culture. au: absorbance units, for optical densities measured at 600 nm.

	**A** $OD_{600} = f(PM)$			**B** $OD_{600} = f(\text{DNA mass})$			C DNA mass $= f(PM)$		
**Replicate #**	**Slope**	**y-intercept**	**R^2**	**Slope**	**y-intercept**	**R^2**	**Slope**	**y-intercept**	**R^2**
1	0.267	0.110	0.954	0.131	0.221	0.867	2.29	-0.832	0.940
2	0.254	-0.017	0.986	0.115	0.286	0.825	1.63	-0.743	0.810
3	0.292	0.176	0.906	0.112	0.353	0.745	2.10	-0.882	0.952
**Average value**	**0.271**	**0.090**	**0.949**	**0.119**	**0.287**	**0.812**	**2.01**	**-0.819**	**0.901**
Standard deviation	0.019	0.098	0.040	0.010	0.066	0.062	0.337	0.070	0.079
**Conversion factor**		**3.36**			**5.98**			**0.904**	
Unit*		mg pellet/au			µg DNA/au			µg DNA/mg pellet	

Table S4. Genes of the *hmu* operon in the Gram-negative bacteria *Bacteroides thetaiotaomicron* VPI-5482. Heme utilization operon and predicted protein function and structure in the reference genome *B. thetaiotaomicron* VPI-5482 (NZ_UYXG01000001.1). Based on the corresponding protein sequences listed in Table S3, protein structures for hmu operon members were predicted using AlphaFold (8). After coloring by B-factor in PyMol (9), highly accurate model regions are shown in red, orange and green correspond to confident regions, while blue regions are modeled with low confidence. OM: outer membrane. IM: inner membrane.

Protein	HmuY	HmuR	HmuS	HmuT	HmuU	HmuV
Expected function	Hemophore, lipoprotein	TonB-dependent OM receptor	CobN/Mg chelatase, putative dechelatase	Permease, periplasmic heme binding protein	ExbB proton channel	ExbD transport protein
NCBI label	Hypothetical protein	TonB-dependent receptor	Cobaltochelatase subunit CobN	Hypothetical protein	MotA/TolQ/ExbB proton channel family protein	DUF2149 domain-containing protein
Accession number	WP_008765021.1	WP_011107297.1	WP_048697403.1	WP_011107294.1	WP_008763053.1	WP_008765016.1
Pfam	14064	00593	02514	unknown	01618	09919
Predicted structure	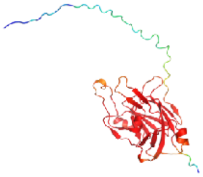	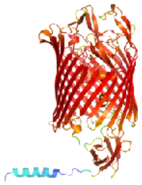	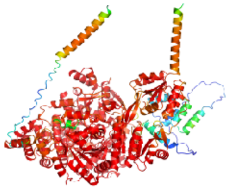	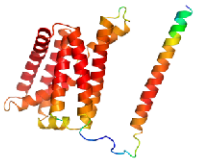	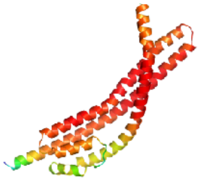	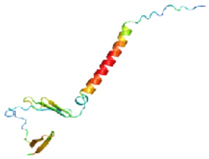
Model quality	87.90	89.89	86.68	87.72	88.30	70.16

Table S5. A complete *hmu* operon is prevalent in the phylum Bacteroidetes. Conservation pattern of 6 genes composing the *hmu* operon across bacterial genomes evaluated by *cblaster* (10) using *B. thetaiotaomicron* VPI-5482 (NZ_UYXG01000001.1) as the reference genome. Hmu protein homology was defined as ≥30% sequence identity. The binary table shows absence=0, presence=1, or two copies=2, and species count for each pattern. Complete *hmu* operons are in bold and account for 2174 (out of 4488) species.

HmuY	HmuR	HmuS	HmuT	HmuU	HmuV	Species count
**1**	**1**	**1**	**1**	**1**	**1**	**2170**
0	1	0	1	1	1	981
0	1	1	1	1	1	385
0	0	0	1	1	1	337
0	1	1	0	1	1	140
1	1	0	1	1	1	84
0	0	1	1	1	1	71
0	1	0	0	1	1	70
0	2	1	1	1	1	39
0	0	1	0	1	1	37
1	1	1	0	1	1	31
1	1	0	0	1	1	23
1	1	1	0	0	1	22
1	2	1	1	1	1	18
1	0	1	1	1	1	17
1	0	0	1	1	1	10
1	1	1	1	0	1	8
0	2	0	1	1	1	7
1	1	1	1	1	1	6
0	1	1	1	1	1	4
1	0	0	0	1	1	3
**2**	**2**	**1**	**1**	**1**	**1**	**2**
**2**	**1**	**1**	**1**	**1**	**1**	**2**
1	2	0	1	1	1	2
1	1	1	1	0	1	2
1	1	0	1	1	1	2
1	0	1	0	1	1	2
0	1	1	1	0	1	2
0	1	0	1	1	1	2
0	1	0	1	0	1	2
1	1	1	0	1	1	1
1	1	0	1	0	1	1
1	1	0	1	0	1	1
1	1	0	0	1	1	1
1	0	1	1	1	1	1
1	0	1	1	0	1	1
1	0	0	1	0	1	1
0	2	1	0	1	1	1
0	1	1	0	0	1	1

REFERENCES

1. **Lewis JP, Plata K, Yu F, Rosato A, Anaya C.** 2006. Transcriptional organization, regulation and role of the Porphyromonas gingivalis W83 hmu haemin-uptake locus. Microbiology-Sgm **152:**3367-3382.
2. **Bacic MK, Smith CJ.** 2008. Laboratory Maintenance and Cultivation of Bacteroides Species. Current Protocols in Microbiology **9:**13C.11.11-13C.11.21.
3. **Mishra S, Imlay JA.** 2013. An anaerobic bacterium, Bacteroides thetaiotaomicron, uses a consortium of enzymes to scavenge hydrogen peroxide. Molecular Microbiology **90:**1356-1371.
4. **Barr I, Guo F.** 2015. Pyridine hemochromagen assay for determining the concentration of heme in purified protein solutions. Bio-protocol **5**.
5. **Marcero JR, Piel III RB, Burch JS, Dailey HA.** 2016. Rapid and sensitive quantitation of heme in hemoglobinized cells. Biotechniques **61:**83-91.
6. **Liu CX, Song YL, McTeague M, Vu AW, Wexler H, Finegold SM.** 2003. Rapid identification of the species of the Bacteroides fragilis group by multiplex PCR assays using group- and species-specific primers. Fems Microbiology Letters **222:**9-16.
7. **Liu X, Olczak T, Guo H-C, Dixon DW, Genco CA.** 2006. Identification of amino acid residues involved in heme binding and hemoprotein utilization in the Porphyromonas gingivalis heme receptor HmuR. Infection and immunity **74:**1222-1232.
8. **Jumper J, Evans R, Pritzel A, Green T, Figurnov M, Ronneberger O, Tunyasuvunakool K, Bates R, Žídek A, Potapenko A.** 2021. Highly accurate protein structure prediction with AlphaFold. Nature **596:**583-589.
9. **Schrödinger L, DeLano W.** 2020. PyMOL. The PyMOL Molecular Graphics System, Version **2**.
10. **Gilchrist CL, Booth TJ, van Wersch B, van Grieken L, Medema MH, Chooi Y-H.** 2021. cblaster: a remote search tool for rapid identification and visualization of homologous gene clusters. Bioinformatics Advances **1:**vbab016.
